# Future land use prediction and optimization strategy of Zhejiang Greater Bay Area coupled with ecological security multi-scenario pattern

**DOI:** 10.1371/journal.pone.0291570

**Published:** 2024-04-18

**Authors:** Shengwang Bao, Wanglai Cui, Fan Yang

**Affiliations:** School of Economics and Management, Zhejiang Ocean University, Zhoushan, China; Jinan University, CHINA

## Abstract

The land use changes driven by human activities press a incredible menace to zonal ecological security. As the most active urban cluster, the uncontrolled expansion of cities in the bay area exerts enormous pressure on the ecosystem. Therefore, from the perspective of ecological conservation, exploring future land use optimization patterns and spatial structure is extremely essential for the long-term thriving of the bay area. On this basis, this research integrated the System Dynamics model (SD) as the quantity forecast model and the PLUS model as the spatial emulation model and established the Land Use/Cover Change (LUCC) Simulation Framework by setting the constraints of Ecological Security Multi-Scenario Patterns (ESMP). By setting four scenarios in future, that is, Business As Usual (BAU), Priority of Ecological Protection (PEP), Balanced Development Scenario (BD), and Priority of Urban development (PUD), this research predicts LUCC in the Zhejiang Greater Bay Area (ZGBA) in 2035 and explored land use optimization patterns. The results indicate that by 2035, under the scenarios of BAU, BD, and PUD, the construction land will observably grow by 38.86%, 19.63%, and 83.90%, respectively, distributed mainly around the Hangzhou Bay Area, Taizhou Bay Area, and Wenzhou Bay Area, primarily achieved by sacrificing ecologically sensitive lands such as forests to achieve regional high economic growth. Under PEP, the growth of construction land retards, and forest experiences net growth (11.27%), with better landscape connectivity and more cohesive patches compared to other scenarios. Combining regional planning and analysis at the city scale, Hangzhou Bay area (Hangzhou, Huzhou, Jiaxing, Shaoxing, Ningbo) can adopt the BD development scenario, while Zhoushan, Taizhou, Wenzhou and Fuyang County of Hangzhou can adopt the PEP development scenario. This research furnishes a novel mechanism for optimizing land use pattern in ecological security perspective and offers scientific guidance for land resource management and spatial planning in ZGBA.

## 1 Introduction

Human activities, represented by the process of urbanization, are continuously changing the land use/cover patterns worldwide and driving a gravely menace to global ES [1]. With the fleeting increase of economy and population, the LUCC resulting from uncontrolled Urban sprawl have become one of the most significant elements threatening the long-term development of ecosystems [2, 3]. From 1970 to 2020, the rate of urbanization in China has risen sharply from around 18% to 64% [4, 5], exerting immense stress on ecosystems and environmental health [6, 7], including biodiversity loss and soil degradation. The bay area represents the most dynamic urban cluster driving economic and innovative development, but in recent decades, rapid urban expansion has led to severe ecosystem deterioration [8]. Therefore, how to safeguard the ecological zone and the LUP has become the most crucial research topics for future sustainable development in the bay scale [8–10].

Predicting the future LUCC by establishing multiple scenarios can provide decision-makers with different possibilities for optimizing land use patterns [11]. The adopted model encompasses both quantitative and spatial dimensions of change [10]. The LUCC quantity simulation models should be able to visually display the anticipated transformations of different land types. Currently, the mainstream quantity prediction models in academia include the Gray Model (1,1), Markov model, Linear model, and others [12]. The Gray Model (1,1) and Linear models treat land use area as a number sequence and perform regression and numerical prediction using formulas [10]. However, LUCC does not necessarily follow linear or exponential trends and is also influenced by policies, society, and other factors [13]. Thus, characterizing LUCC solely based on mathematical models not only results in lower accuracy but also lacks the ability to set scenarios. The Markov model, which analyzes the direction and speed of land class transitions by extracting LUCC between two periods, offers higher precision and has become one of the main quantity prediction models in LUCC spatiotemporal simulations [14]. However, the Markov model heavily relies on the time step and lacks simulation coherence [15, 16]. Additionally, according to the Markov model, adjusting the future land demand by modifying transition probabilities between different land types has becomed the main approach for setting different development scenarios in a region [17]. However, this method heavily relies on subjectively distinguishing the direction and rate of land use transitions, making it daunting to fully understand policy and economic factors [10, 17–19]. LUCC is driven by the interaction of multiple elements, such as socio-economic and natural elements [13]. Therefore, compared to other quantity models, the SD model can consider various influencing factors, including socio-economic aspects, and establish a complex interactive structure of LUCC that suits the practical status of development of bay area, setting different paths for optimizing land use pattern [10, 20, 21].

Spatial simulations of LUCC focus on the performance of LUCC in different spatial-temporal contexts and geographical locations, revealing the internal transformation mechanisms [22]. CA-Markov, FLUS, and CLUE-s are usually utilized as the spatial prediction models. However, CA-Markov struggles to consider the influence of environmental factors on LUCC and lacks the ability to integrate predictions from other quantitative models [14]. The CLUE-s model focuses on urban boundary expansion and performs well at a small regional scale but is less suitable for simulating the bay area at a larger scale [23]. Nonetheless, the FLUS model heavily relies on base-period land use data and has limited model capacity [9, 22, 24]. The PLUS model can more effectively uncover the mechanisms of LUCC and demonstrates higher accuracy in simulations [22, 25]. Considering simulation accuracy, bay area characteristics, and data sources, the SD-PLUS model are combined to be more appropriate for exploring LUP in the bay area.

Existing models often use water as a constraint for future LUCC simulations, overlooking the function of ecosystem services of protecting ES [24]. ESP is regard as a comprehensive eco-network and aims to identify key areas for ecological resource protection, thus serving as practical ecological constraints [17]. Over the past decade, ESP has converted its emphasis from Biodiversity Conservation, Landscape and Ecology to Urban Expansion Management Strategy [26, 27]. Increasingly, studies are combining LUCC with ESP, using ESP as a constraint to simulate future LUCC and explore long-term land use policies in the region [8, 17, 26, 28]. However, traditional ESP lacks the ability to set scenarios and form a complete system [16]. Consequently, to establish ESMP is extremely vital to the flourish and ES of bay area [17] through ecological sources identification, resistance surfaces construction and ecological corridors extraction [29, 30]. In ecological sources identification, researchers focus on regional LUCC and the assessment of ecosystem service values [31, 32], striving to balance multiple interests in ecological conservation [17, 33]. Additionally, MCR has been broadly utilized to connect ecological source areas while considering landscape heterogeneity [8]. By combining quantitative assessment of ecosystem services and MCR to construct a ESMP, it will furnish a scientific reference for decision-maker in enacting urban spatial land planning and management strategy.

The bay area serves as a prominent symbol of world-class coastal cities and has become an important experience for countries worldwide to develop open economies and establish strategic advantages [8, 10]. As the region with the most intense and dramatic urbanization process in China [34–36], ZGBA exhibits typical land use spatial characteristics, with the implementation of multiple national strategies [37]. *The Fourteenth Five-Year Plan for Economic and Social Development of Zhejiang Province and the Vision for 2035* explicitly states the goal of constructing a modern bay area that leads the future. Along with rapid population growth and increased resource consumption in the ZGBA, ecological and environmental issues such as intensified conflicts between land development and ecology protection, imbalanced land use structure, and severe land loss have emerged [17, 36]. Therefore, it is fully vital to maintain and promote the integrality of the total ecosystem health in the region for future development.

This study proposes an integrated ESMP-SD-PLUS model that combines quantitative and spatial constraints to simulate LUCC in the ZGBA by setting ESMP in 2035 on the basis of land use data from 2005 and 2020. The main purpose of our research are: (1) to quantitatively evaluate the value of ecosystem services to identify ecological source areas; (2) to calculate ecological resistance surfaces and extract ecological corridors to establish ESMP constraints; (3) to couple the SD model and PLUS model to emulate LUCC in 2035 under the spatial constraints of ESMP. The research is helpful to explore strategies for ecological land protection, LUP, and the conservation of ES in the bay area, while providing more scientific recommendations for land use planning in different cities within the ZGBA. This research aims to put forward differentiated land management strategies in ZGBA from the perspective of ES, and to provide scientific references for relevant decision-making on ecological protection and sustainable development.

## 2 Research area and materials

### 2.1 Study area

**Fig 1:**
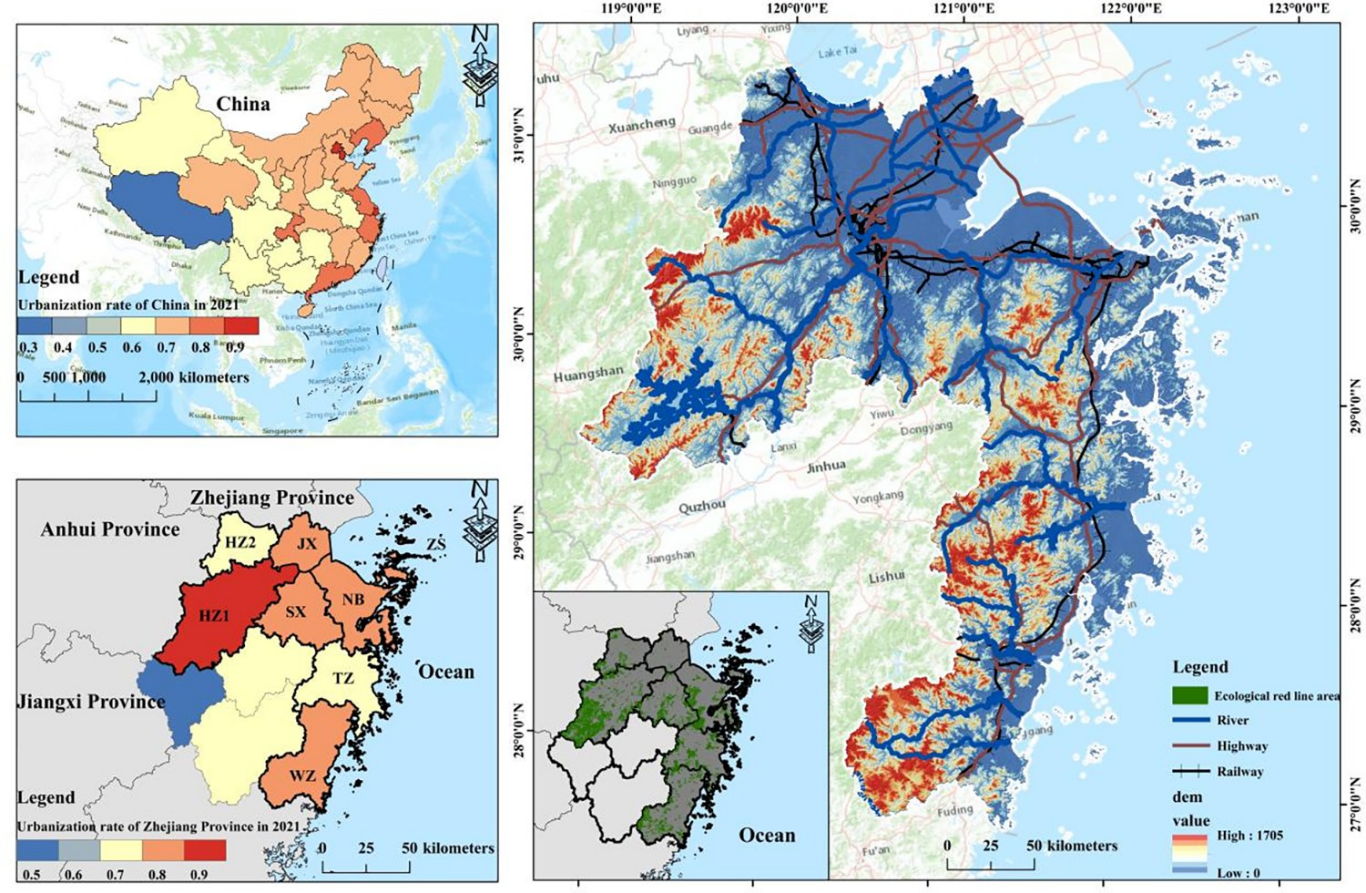
Study area.

ZGBA (119°03′-123°10′E, 27°02′-31°11′N) is located on the southeastern of the Yangtze River Delta urban agglomeration. It is centered around the Hangzhou Bay Economic Zone and encompasses other bay areas such as Wenzhou Bay, Sanmen Bay, Taizhou Bay, Yueqing Bay. The region consists of eight cities: Hangzhou (HZ1), Huzhou (HZ2), Jiaxing (JX), Ningbo (NB), Shaoxing (SX), Taizhou (TZ), Wenzhou (WZ), and Zhoushan (ZS) (Fig 1). The total land area is approximately 66,255.38 km^2^, accounting for 64% of Zhejiang Province. ZGBA has a subtropical monsoon climate with an average annual temperature of 9–21°C and an average annual precipitation of about 14,771 mm. With a coastline of 6,696 km, ZGBA has the longest coastline in China. It is also home to 4,350 islands, accounting for about 40% of China. ZGBA is the fastest-developing region in China’s modernization process and the largest urban clusters of most dynamic economic in the world. As of the end of 2021, the urbanization rate in ZGBA has reached 74.3%, with a GDP of 6,457.458 billion yuan, accounting for approximately 5.6% of the national total with only 0.7% of the total national area. The population density in ZGBA is 807 people/km^2^, and the economic density is 9,746.22 million yuan/km^2^. However, the economic density in the region is only 8.9% of the Tokyo Bay Area, 18.6% of the New York Bay Area, 24.2% of the San Francisco Bay Area, and about 40.6% of the GBA, indicating relatively low efficiency of land utilize. The rapid development of ZGBA has also generated incremental conflicts between land supply and demand, imbalanced land use structure, and severe loss of ecological land, posing significant challenges to protect es in the bay area.

### 2.2 Data sources

The data used in the research can be categorized based on their purposes: Ecosystem Services Assessment, Resistance Surface Construction (RS), Driving Factors of LUCC Simulation (DF), and Land Use Demand (LD). The concrete data are outlined in the Table 1.

**Table 1:** Data source and application.

Data	abbreviation	resolution	sources	application	time
Land use and land cover change	LUCC	30m	LUCC CLCD [41]	CS, HQ, WY, RS	2000–2020
Digital Elevation Model	DEM	30m	Geospatial Data Cloud (https://www.gscloud.cn/)	SDR, RS, DF	2020
Slope	SLOPE	30m	Extracted by DEM	RS, DF	2020
Normalized Vegetation Index	NDVI	30m	National Earth System Science Data Center (https://www.geodata.cn/)	RS, DF	2020
Population	POP	1000m	Word population (https://hub.worldpop.org/)	RS, DF	2020
Precipitation	PRE	1000m	National Earth System Science Data Center (https://www.geodata.cn/)	WY, RS, DF	2020
Temperature	TEM	1000m	National Earth System Science Data Center (https://www.geodata.cn/)	RS, DF	2020
Distance to Road (national road, provincal road, highway, railway)	DN, DP, DH, DR	Vector	Openstreetmap (https://www.openstreetmap.org)	HQ, RS, DF	2020
Gross Domestic Product	GDP	1000m	pixel level of GDP [42]	RS, DF	2020
Rainfall Erosivity	R	1000m	Erosivity mapping [43]	SDR	2020
Evapotranspiration	ETO	1000m	TPDC (https://data.tpdc.ac.cn/) [44]	WY	2020
Soil Data (Maximum root depth、PAWC、Soil erodibility)	-	-	Harmonized World Soil Database (https://www.fao.org/)	WY, SDR	2020
Net primary product	NPP	1000m	MODIS NPP product	RS, DF	2020
Biophysical table, sensitivity, threat	-	-	Defined as the previous literature and the InVEST User Guide	CS, HQ, SDR, WY	2020
Carbon Pool	-	-	Ecosystem Carbon Storage in Hangzhou Bay Area Based on InVEST and PLUS Models [45]	CS	
Ecological Red Line	-	Vector	Zhejiang Province Government		
Socialeconomic data	-	-	China Statistical Yearbook, Zhejiang Provincial Statistical Yearbook, Gridded datasets for population and economy under Shared Socioeconomic Pathways [40]	LD	2000–2020

(1) The land use classification system is classified into six categories, which is cultivated land, forest, meadow, water, construction land, and unused land, based on the *Classification Standards for Land Use Status in China* (GB/T 21010–2017) [38]. (2) Four types of ecosystem services were evaluated by InVEST model, based on previous research on ecosystem services assessment [8, 17, 31, 32, 39]. Different datasets were used to assess different ecosystem services, as indicated in Table 1 under the headings CS, HQ, WY, and SDR. (3) Thirteen resistance factors, including LUCC, were incorporated to calculate the ecological resistance surface. Previous researches have displayed that land use have strongly impacts on ecological resistance [8], so including LUCC as a resistance factor improves the scientific basis of resistance surface construction. See Table 1 under the heading RS. (4) In the process of simulating future LUCC, factors such as climate, environment, and human interference that influence LUCC changes need to be considered. DEM, NDVI, population, and other factors were used as driving factors for LUCC simulation, as shown in Table 1 under the heading DF. (5) Social and economic data were used to simulate land demand in ZGBA using system dynamics. The data sources are mainly extracted from the China Statistical Yearbook and the Zhejiang Statistical Yearbook. See Table 1 under the heading LD for more details.

Population and GDP data for different scenarios in Zhejiang Province were obtained from Jiang’s work [40]. The population and GDP for the years 2021–2035 under the SSP1, SSP2, and SSP5 scenarios were calculated based on the average proportions of population and GDP in the ZGBA from 2000 to 2020. To analyze past, present, and future climate changes, the World Climate Research Program (WCRP) introduced the Coupled Model Intercomparison Project Phase 6 (CMIP6) in 2021 [20], which sets five shared socioeconomic pathways (SSP1-5). SSP1-2 and SSP5 were selected as the socioeconomic baselines for the scenario of Business As Usual (BAU), Priority of Ecological Protection (PEP), Balanced Development (BD), and Priority of Urban development (PUD). The resolution was adjusted to 30m using resampling. Additionally, the coordinate system WGS-UTM-50N was used and spatially missing values were filled using kriging interpolation.

## 3 Methodology

### 3.1 The construction of ESMP-SD-PLUS model

This study proposes an integrated LUCC simulation model, the ESMP-SD-PLUS model, which combines the ESMP with SD model and PLUS model. It employs a "top-down" and "bottom-up" approach to predict future LUCC in ZGBA under ESMP. To achieve spatially consistent ecological constraints, the ESMP incorporates ecosystem services assessment, overlay analysis, and identify ecological source areas by MSPA. Differentiation ecological constraints is constructed by MCR model. The land demand under the BAU is calculated of Markov prediction module of PLUS model. The land demand under the SSP1, SSP2, and SSP5 scenarios is calculated using the SD with the PEP, BD, and PUD scenarios. Merely the ecological redline areas are used as ecological constraints in BAU. To ensure connectivity between ecological source areas, additional ESPs with different constraint scales are included in the PEP, BD, and PUD scenarios, in addition to the ecological redline areas. The PLUS model is selected to predict land use in ZGBA in 2035. Based on the analysis of LUCC at the city scale and landscape pattern index analaysis, policies of LUP are proposed to enhancing the practicality and scientific basis of this research.

### 3.2 The construction of ESMP

#### 3.2.1 Ecological sources identification of ZGBA

**Fig 2:**
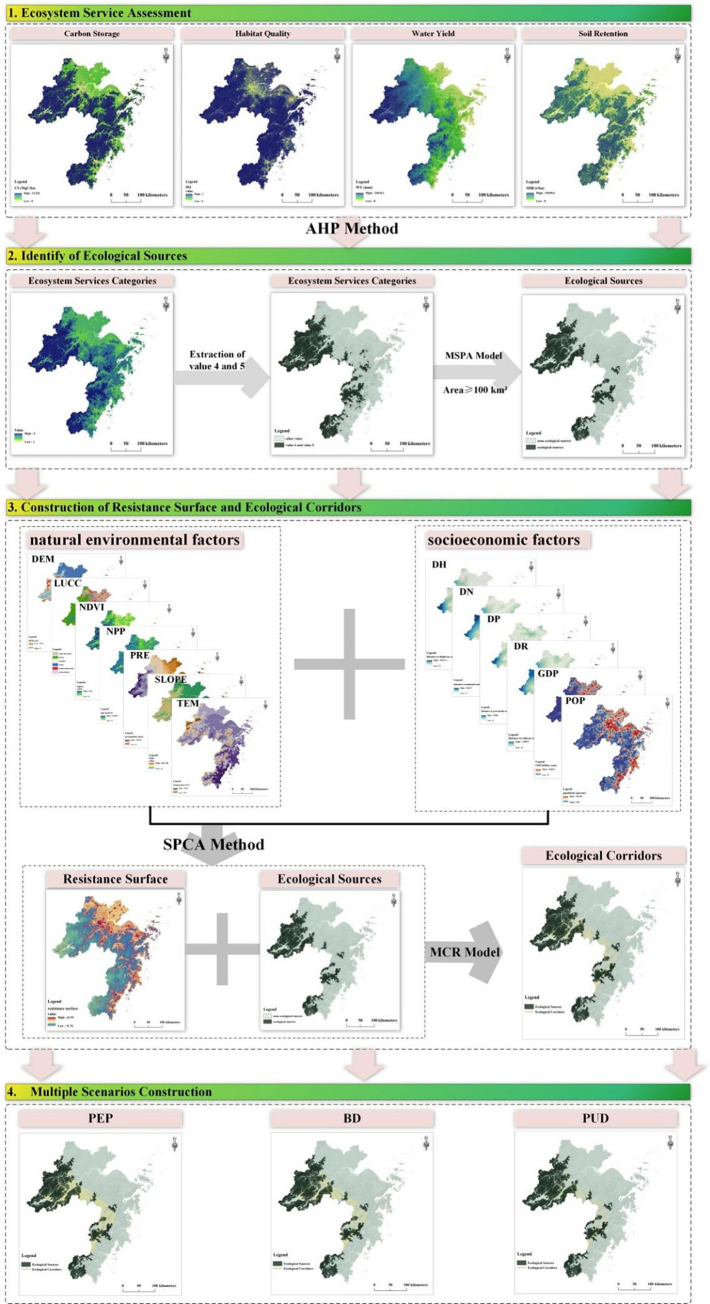
The construction of ESMP.

Ecological sources are crucial regions for maintaining the monolithic stability and consecutiveness of regional ecosystems, and they play a significant role in ensuring ES [46]. By assessing the ecosystem services and utilizing MSPA model, ecological source areas can comprehensively reflect the ecological attributes and courses of ZGBA [24, 47]. The InVEST model was used to assess ecosystem services in ZGBA, including CS, HQ, WY and SDR. The Analytic Hierarchy Process Method was employed to calculate the weights of different ecosystem services, and the results were overlaid. The natural breakpoints method was applied to sort the overlaid results into 5 levels, allowing for an evaluation of their importance, where higher values indicate greater ecological significance. Subsequently, the MSPA was applied to identify core patches in the fourth and fifth levels, and core patches greater than 100 km^2^ were considered as ecological source areas. The process is illustrated in Fig 2. Ecosystem services are calculated as follows:

Carbon Storage, CS

CS can be classified into four components: aboveground and belowground biomass carbon pools, soil carbon pool, and dead organic carbon pool [45]. The formulation is shown as follows:

$$C_{total}=C_{above}+C_{below}+C_{dead}+C_{soil}$$ (1)

Among them, C_above_ refers to the carbon in lifes above the ground surface. C_below_ refers to the carbon stored in the live root systems of plants. C_dead_ represents the carbon stored in litter, standing dead trees, or deadwood, mainly referring to the litter carbon pool. C_soil_ generally means the organic carbon content soil. The parameters for carbon density can be found in Appendix S1.1 in S1 Appendix.

(2) Habitat Quality, HQ

HQ refers to the ability of an ecosystem to provide sustainable conditions for individuals and populations [48, 49]. It typically decreases with increasing land use intensity in the surrounding areas [50]. The formulation is shown as follows:

$$Q_{\mathrm{xj}}=H_{j}(1‐(\frac{D_{\mathrm{xj}}^{z}}{D_{\mathrm{xj}}^{z}+k^{z}}))$$ (2)

Where, *Q*_*xj*_ represents the habitat quality of grid x for land use j, *H*_*j*_ means the habitat suitability of land use type j, *D*_*xj*_ represents the threat level of land use type grid x, Z and K is the normalization and scaling constant, respectively. The parameter settings are based on the model User’s Guide [51], relative literature [8, 17, 24], and Specialist Consultation. The specific calculation formulas and parameter settings can be found in Appendix S1.2 in S1 Appendix.

(3) Water Yield, WY

WY is closely related to climate factors, underlying surface conditions, human activities, and plays a crucial role in water supply, regulating runoff, and soil conservation in the ecosystem and watershed water cycle [51].


$$Y(x)=(1‐\frac{AET(x)}{P(x)})\times P(x)$$ (3)

In the equation, *Y*(*x*) and *P*(*x*) represents the water yield of grid x (mm) and the precipitation in year t of grid x (mm),respectively. *AET*(*x*) displays the actual evapotranspiration of grid x (mm). Specific parameter values can be found in Appendix S1.3 in S1 Appendix.

(4) Sediment Delivery Ratio

Sediment Delivery Ratio (Soil Retention) is an essential function of ecosystem services, as it contributes to increasing water supply, reducing soil erosion, and maintaining regional ES [39]. The Revised Universal Soil Loss Equation (RUSLE) was used to estimate soil conservation capacity [17]. The specific calculation formula is shown below:

SDR=R×K×LS×(1−C×P) (4)

R and K is the rainfall erosivity factor and soil erodibility factor, respectively. LS is the slope length and steepness factor. Specifically, L represents the slope length factor, S represents the slope steepness factor, which are two factors that reflect the influence of terrain on soil erosion. C and P represents the cover management factor and support practice factor. The specific parameter settings can be found in Appendix S1.4 in S1 Appendix.

#### 3.2.2 Resistance surface construction and ecological corridors extraction

The construction of ecological resistance surface can reflect the resistance during species migration process [52]. To avoid redundancy in spatial information, Spatial Principal Component Analysis Method is used to derive the weights for the ecological resistance surface. The specific formula and results can be found in Appendix S3 in S1 Appendix.

MCR is widely applied to extract ecological corridors in the region scale by constructing the resistance surface of species migration from one source to another [53]. In this research, we used ArcGIS 10.8 software to extract the ecological corridors. The formula for the MCR model can be found in Appendix S2 in S1 Appendix.

#### 3.2.3 Four development scenarios of ZGBA

**Fig 3:**
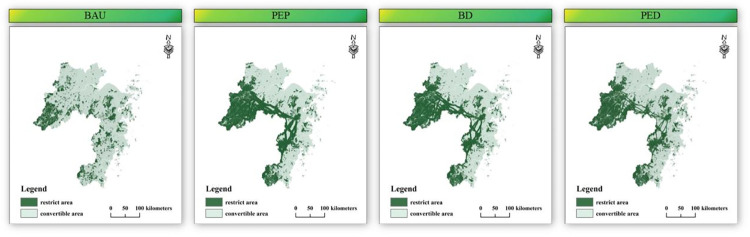
Different development scenarios combined with ESMP.

Based on the range and buffer width of ecological corridors, three differentiation widths of ESMP were constructed as spatial constraints (Fig 3), namely Optimal ESP (OESP), Satisfactory ESP (SESP), and Bottom-line ESP (BESP). Studies have shown that ecological corridors effectively enhance connectivity between different ecological patches and serve as pathways for species migration [8, 17, 53]. Establishing ecological corridors with a certain width range will contribute to the protection of ecosystems and biodiversity, and serve as ecological buffer zones in the ZGBA. According to the *Zhejiang Greater Bay Area Construction Plan* [54] and referencing relevant studies [8, 10, 17, 24], we established ecological corridor buffer zones of 1200m, 2400m, and 3600m as restrictions for the BESP, SESP, and OESP, respectively.

Considering the different future development scenarios of the bay area, four scenarios were designed to simulate future LUCC: BAU, PUD, PEP, and BD. The ecological redline area refers to the areas which must be strictly protected by mandatory measures, serving as the bottom line for safeguarding and maintaining global and regional ES. Therefore, the ecological redline area is the basic constraint for all scenarios, and the only constraint for the BAU scenario. The remaining three development scenarios are combined with their corresponding ESMP to construct ecological constraint areas, aiming to mitigate the impacts of human activities. To align with the development strategies of different scenarios, we supplemented the BESP, SESP, and OESP as ecological restrictions for PUD, BD, and PEP.

### 3.3 Construction of future development scenarios based on system dynamics

**Fig 4:**
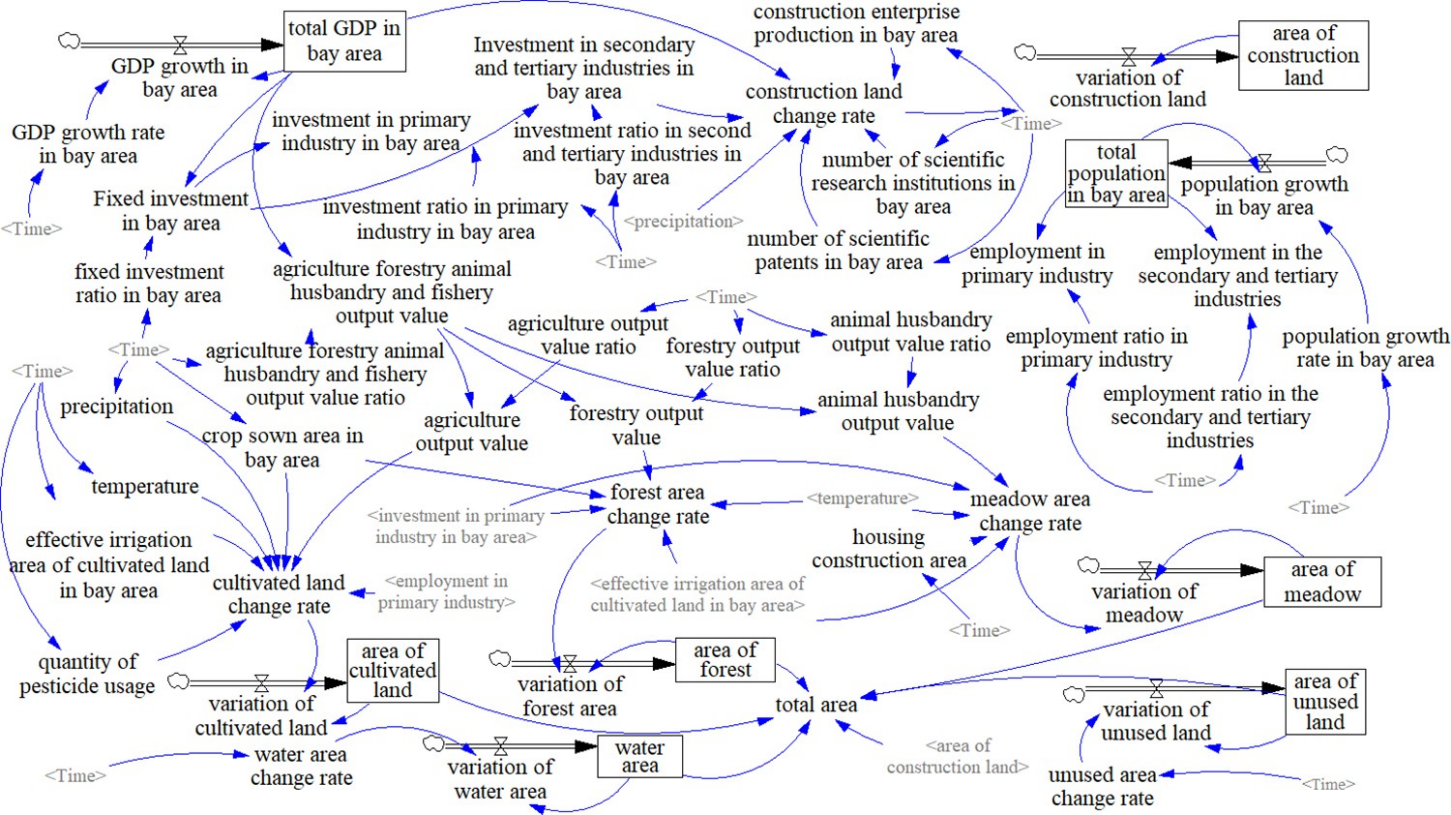
The structure of SD model.

Based on the research achievements of Jiao [10] and Wang [20] and considering the actual situation of ZGBA, this study constructs an SD model structure consisting of five subsystems of economic, population, climate, social, and land use (Fig 4). Economic development influences the value added of primary industries and investments in secondary and tertiary industries, thereby affecting the shifts in cultivated land, forest, meadow, and construction land. The population subsystem reflects the population employment structure. An increase in employment in the primary industry often results in an increased demand for cultivated land, thereby affecting the structure of LUCC. The climate subsystem mainly includes climate variables such as precipitation and temperature, which are the primary natural environmental factors that directly affect LUCC, particularly for ecologically sensitive land types such as meadow and cultivated land. The social subsystem mainly reflects the influence of various social actors on LUCC. For example, the output value of construction enterprises directly affects changes in construction land, and the irrigation rate of productive farmland affects cultivated land. The land-use subsystem mainly considers the most direct reflection of LUCC under the combined influences of various subsystems. Based on social and economic data, natural environmental data, and land-use data, the SD model structure is constructed by Vensim PLE software (Fig 4). The period from 2000 to 2020 is used as the validation of SD model, and the simulation period is from 2000 to 2035, with one year as the time step. During the validation stage, historical data from 2000 to 2020 is used for simulation and compared with real data. Considering the different choices and scenarios for the future development of ZGBA, we have set different development scenarios of future land use demand prediction.

#### 3.3.1 Validation of SD model

Before simulating the future land demand in the ZGBA in 2035, it is necessary to validate the effectiveness of the SD. Using indicator data from 2000 to 2015, we simulated the areas of different land use types in 2020 and compared them with the actual data in 2020. The land use types average errors from 2000 to 2020 were as follows: cultivated land (-1.37%), forest (0.24%), meadow (-0.69%), water (0.0003%), construction land (-0.13%), and unused land (-0.0069%). The specific results are shown in Appendix S4 in S1 Appendix.

#### 3.3.2 Model parameter setting under different development scenarios

Considering the different scenarios for the future development, and referring to the SSP of CMIP6, we have set 3 scenarios for the future development of ZGBA: PEP, BD, PUD. These scenarios are based on the population and GDP data derived from the research results of Professor Jiang’s achievements of SSP1, SSP2, and SSP5. With the 2020 as the baseline year and by inputting the scenarios setting parameters, we simulated the land use demand in the future. In addition, the BAU scenario uses land use data from 2005 and 2015 predicted by the Markov model. For other parameters, please refer to Appendix S5 in S1 Appendix.

### 3.4 The spatial-temporal simulation of LUCC in ZGBA in 2035

#### 3.4.1 Spatial simulation of land use

The Patch-based Land Use Simulation model (PLUS) [22] uses the random forest classification algorithm to extract land use expansion at different time periods and analyze the contribution of driving factors to generate land use transition probabilities [22, 25]. The model is selected to simulate LUCC by setting land use transition matrices, land demand, and neighborhood weights to simulate future LUCC.

#### 3.4.2 The selection of driving factors

12 driving factors of natural environmental and socio-economic factors have been selected as the determinants of LUCC. The specific selection of factors is detailed in Appendix S6.1 in S1 Appendix.

#### 3.4.3 Parameters of PLUS model

By using the land type transition matrix, we can determine whether different land types can be transfered into each other, and the magnitude of neighborhood weights represents the dispersal capability of land types. Taking the 2005–2020 land use transition matrix as a reference, appropriate adjustments are made based on the scenarios. The neighborhood weights are determined based on the proportion of each land type area for the total area of ZGBA in 2020. For more details, please refer to Appendix S6.2 in S1 Appendix.

#### 3.4.4 The validation of simulation result of LUCC

Before predict the future LUCC, to validate the effectiveness of PLUS is extremely necessary. Using the land use rensing image from 2010 and 2015, we simulated the LUCC in 2020 and compared it with the actual situation. Please refer to Appendix S6.3 in S1 Appendix for detailed information. Previous studies have shown that the kappa coefficient is an effective measure to validate the reliability of simulation results [22, 55]. Therefore, the kappa coefficient was used to validate the simulated results of our model. The results showed a kappa coefficient of 0.92 and an overall accuracy of 0.95, indicating excellent performance and reliability of the results.

### 3.5 Landscape pattern index changes under different scenarios

Landscape pattern index are powerful tools for analyzing the spatial structure and characteristics of LUCC [10]. Referring to studies conducted in similar bay areas [10, 56], eight landscape pattern index were selected to capture the spatial characteristics of LUCC in the ZGBA from four dimensions: aggregation, shape, fragmentation, and diversity. The indices for landscape aggregation include Landscape Juxtaposition Index (LJI) and Contagion Index (CONTAG); for landscape shape, Landscape Shape Index (LSI) and COHESION; for landscape fragmentation, Landscape Pattern Index (LPI) and DIVISION; and for landscape diversity, Shannon’s Diversity Index (SHDI) and Shannon’s Evenness Index (SHEI). The descriptions and formulas of these indices can be found in Appendix S7 in S1 Appendix.

## 4 Result

### 4.1 Forecast of future land demand based on SD model

**Fig 5:**
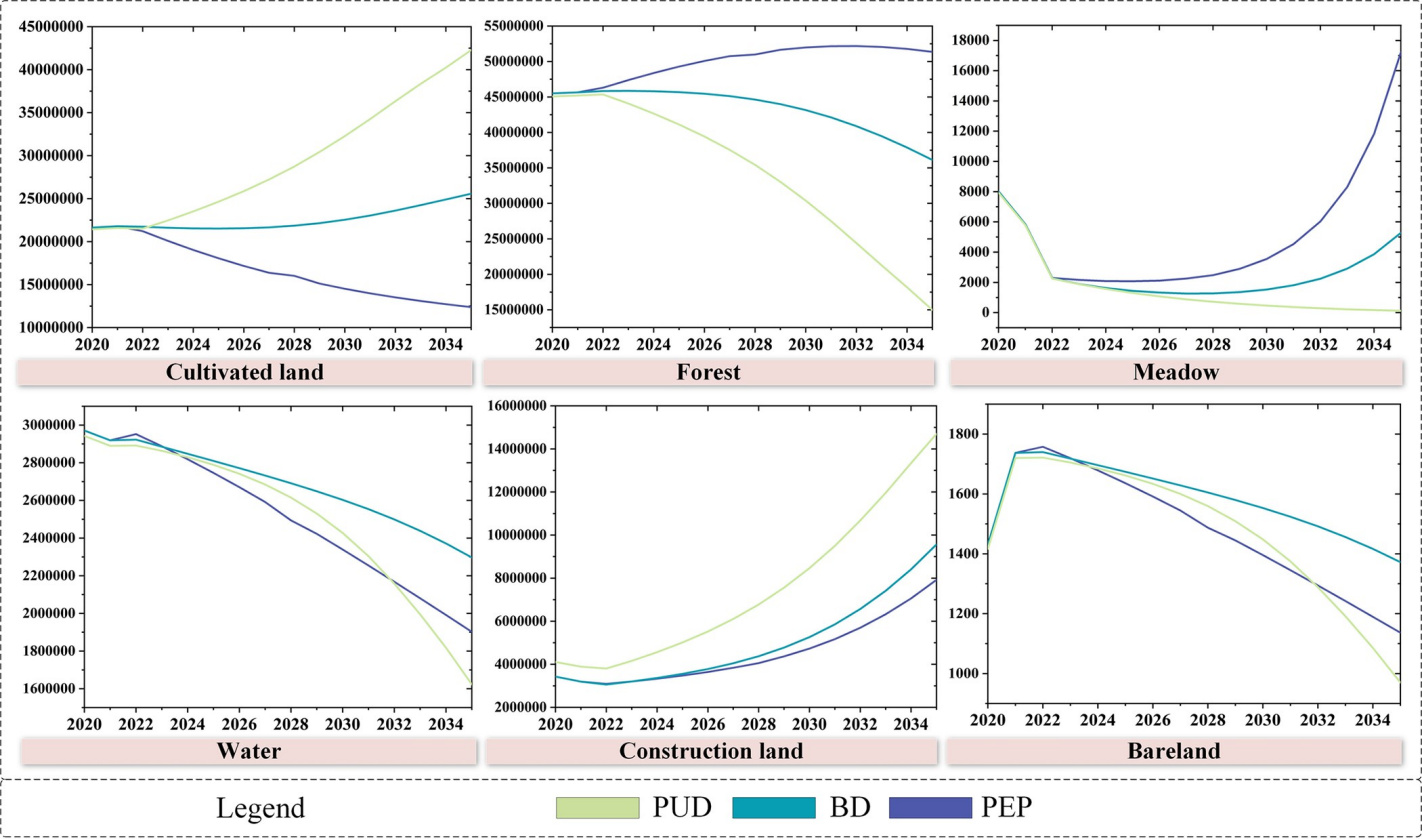
Land demand prediction.

Based on the social and economic data from 2000 to 2015, variables and equations has been set to predict the land demand changes of 2000–2020. The simulated land demand were compared with the actual land use situation, and the model showed good performance. From 2000 to 2020, the parameters for the PEP, BD, and PUD scenarios were set by SD model to predict the land demand in 2035, and the predicted results are shown in Fig 5. The results indicate that the land use demands vary under different scenarios. In all three scenarios, the area of construction land increases, while the area of water decreases. The trend in the change of cultivated land is consistent with that of construction land, but it is opposite to the changes in forest and meadow. This is cheifly because of the high concentration and influx of population resulting from economic development, which requires more cultivated land to meet the demands of the increasing population, leading to the conversion of more forest and meadow into cultivated land. In all three scenarios, water area and unused land area are in the decreasing trend. Under PUD, the degradation of forest and meadow is severe, and there is the highest growth in cultivated land and construction land. This scenario also has the highest level of development in water and unused land.

### 4.2 Future land use in each scenarios in2035

#### 4.2.1 Spatial-temporal simulation of LUCC in ZGBA

Through the ESMP-SD-PLUS model, LUCC under four scenarios in 2035 was simulated, and the expansion under each scenario was compared, specifically the expansion from 2020 to 2035. At the scale of the Bay Area, the urbanization process shows significant differences, which is manifested by the varying growth of construction land. As to the next 15 years, the scenarios of BAU, BD, and PUD show increases in construction land by 38.86%, 19.63%, and 83.90%, respectively. In the PEP scenario, the construction land remains relatively unchanged compared to the current situation. The drastic increase of construction land under PUD approaches the urban sprawl limit of ZGBA under the spatial restriction of the BESP. In PUD scenario, the mean annual increase rate of construction land is 5.59%, exceeding that of 2005–2020 (2.80%). Meanwhile, in order to match the economic development and urbanization, the PUD scenario shows an increase in cultivated land area by 39.65%, mainly achieved through encroachment on forest for expansion. In the BAU and BD scenarios, the mean annual increase rates of construction land are 2.59% and 1.31%, respectively. Only the PEP scenario achieves a net increase in forest (11.27%), while forest areas decrease by 2.29%, 14.56%, and 33.47% in the BAU, BD, and PUD scenarios, respectively. The results indicate that only the PEP scenario effectively implements the "returning farmland to forest" policy to maximize the prevention of ecological land loss.

**Fig 6:**
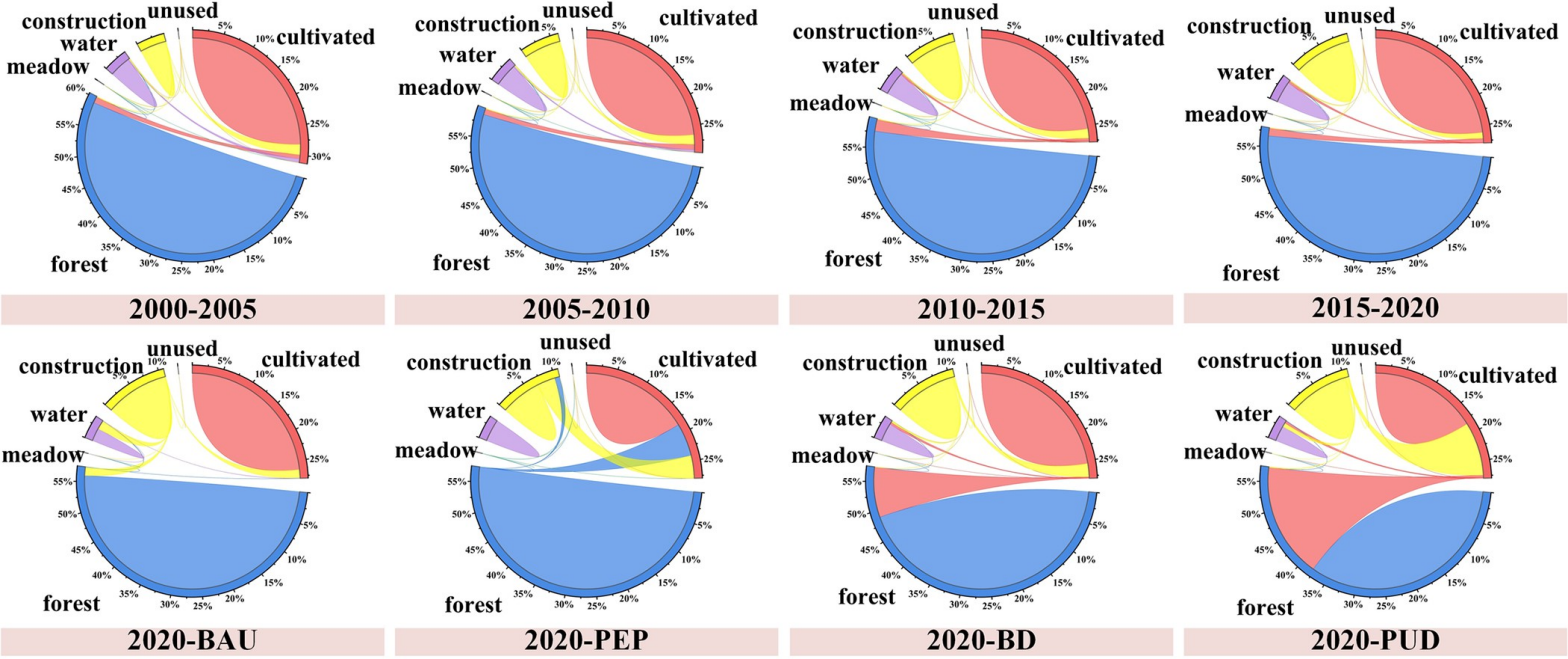
Land transfer string picture.

To illustrate the impact of ESMP scenario constraints on LUCC structure, we compared the land use transitions from 2000 to 2020 (with a 5-year interval) and the different development scenarios from 2020 to 2035 (Fig 6). The results indicate that the construction land sprawl primarily comes from encroaching on cultivated land under PUD. To ensure high-quality development, the increase of cultivated land chiefly results from the "conversion of forestland to cultivated land," which is a direct reason of rapid ecological land reduction. In addition, in the PEP scenario, cultivated land is primarily transformed into forest and construction land, with LUCC transitions generally opposite to the PUD scenario.

**Fig 7:**
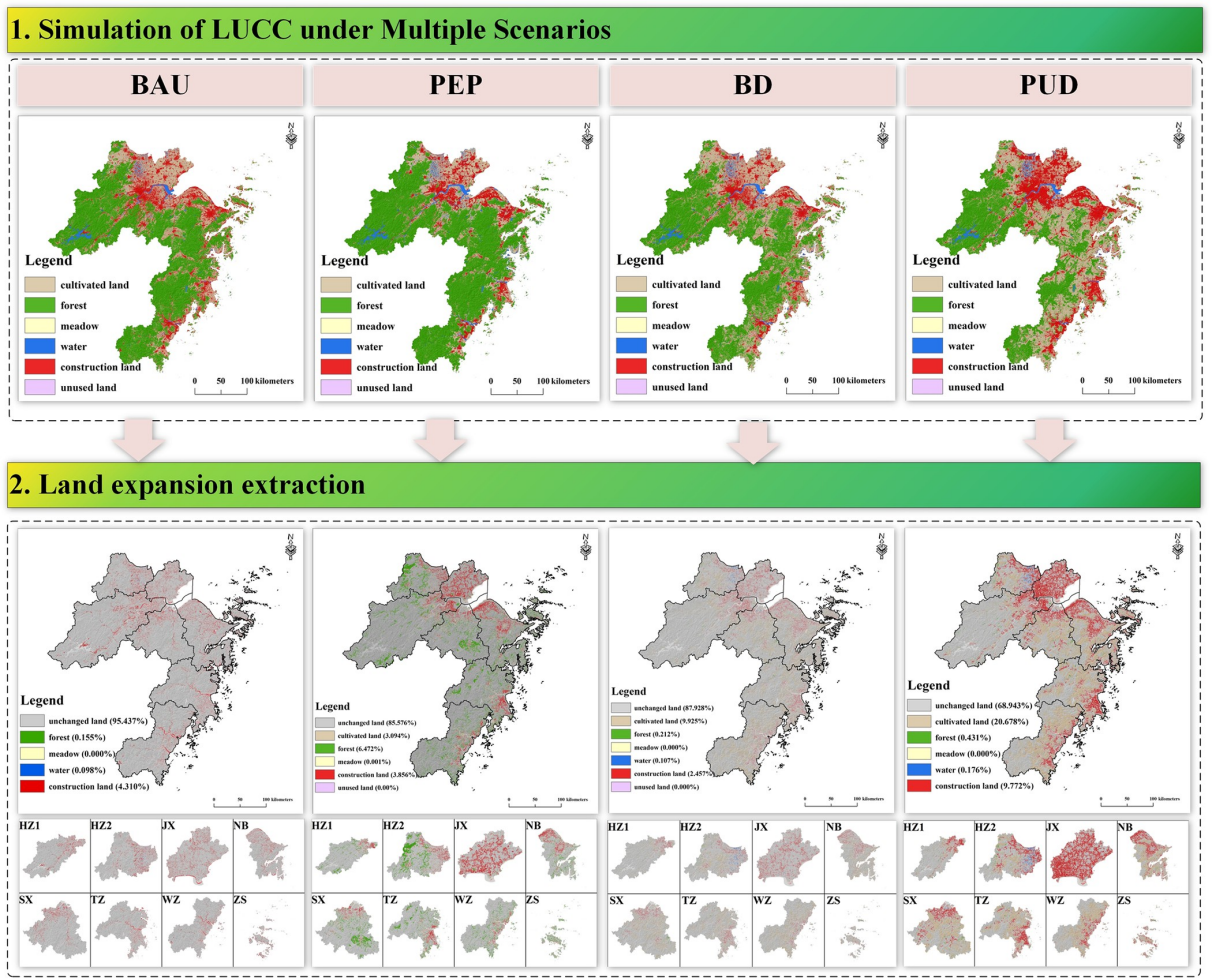
Spatial distribution of LUCC in future.

With regard to spatial distribution, construction land expansion is mainly concentrated around the Wenzhou Bay, Taizhou Bay, and Hangzhou Bay (Fig 7). Such result aligns with the spirit of the 15th Party Congress of Zhejiang Province, which emphasizes accelerating the development of a world-class bay, aiming to develop the coastal modern industrial belt along the Hangzhou Bay, WZ and TZ by establishing a coastal corridor development model. Furthermore, JX, as an important city connecting HZ1 and Shanghai, becomes a focal point for the future construction of the Hangzhou Bay and even the ZGBA.

#### 4.2.2 Analysis in a city scale

**Fig 8:**
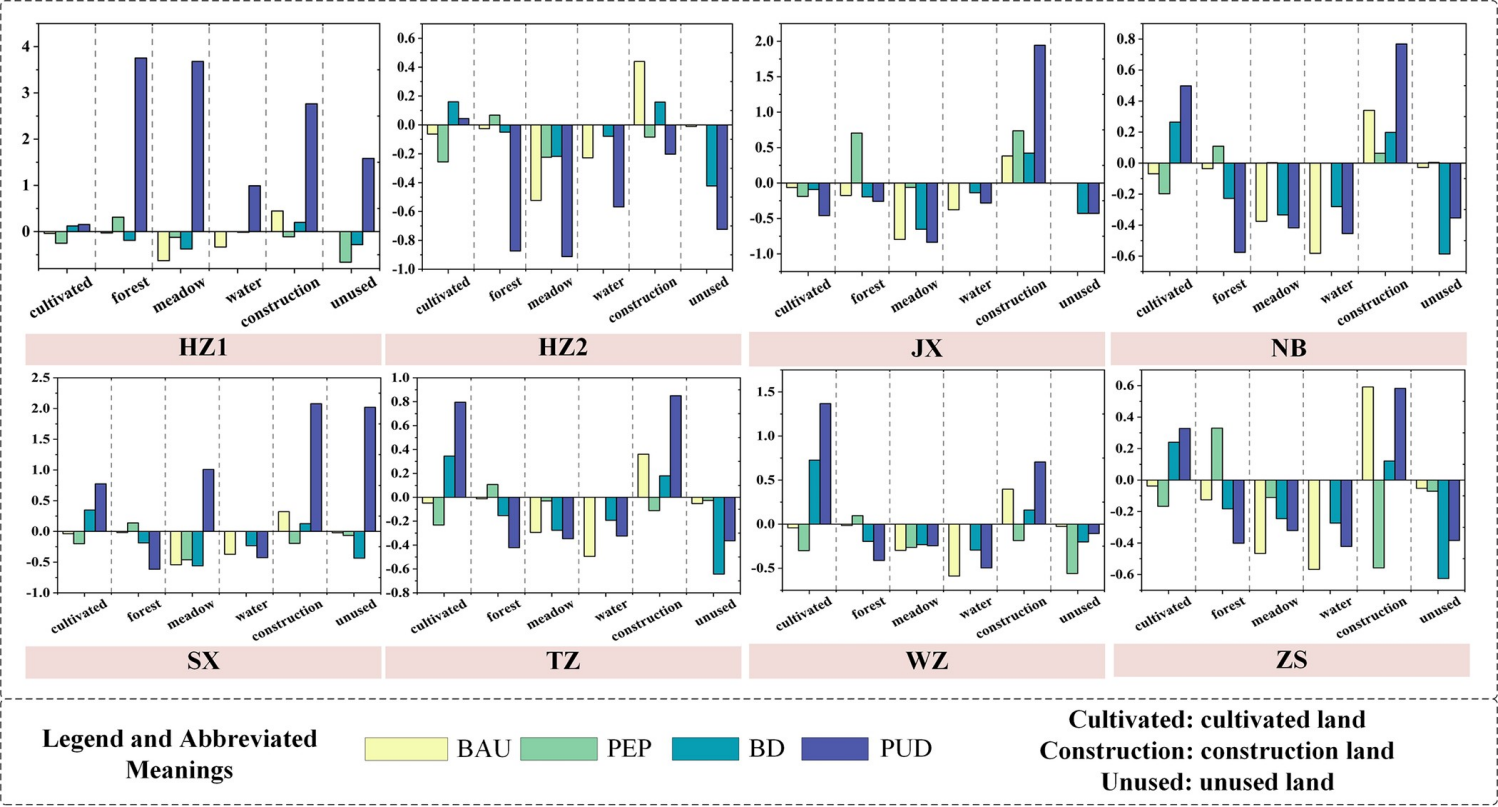
Change of land area in each city.

The areas of land use in each cities of ZGBA are shown in Fig 8. Overall, the area of construction land of ZGBA appears an increasing tendency, except for ZS in the PEP scenario, which is allied to the composition of ZS as an island county primarily reliant on port and fishing economies. The increase of construction land is allied to the low proportion of construction land (10.86%) and the availability of abundant land resources for development. Therefore, in the PUD scenario, which approaches the development limit of the Bay Area, the expansion of residential areas and the development of industrial and commercial clusters are the main types of construction land expansion. There is rapid expansion of cultivated land, but development based on the premise of ecological land destruction leads to imbalanced structure of land use and development efficiency reduction. Therefore, improving the effective productivity of cultivated land and promoting the clustering of the primary industry will effectively address this situation. NB, TZ, and WZ are the main cities where forest decreases and cultivated land increases, which aligns with construction land expansion spatial distribution. Additionally, the area of meadow shows significant fluctuations under different scenarios, influenced by its own ecological sensitivity, without a consistent pattern. Attention and protection are needed in the future development process. The boost rate of construction land under PEP significantly decreases and is the lowest among the four scenarios, which demonstrates that the set ESMP plays a significant role. Compared to the PUD and BD scenarios, the rates of construction land under PEP decrease by 46.13% and 17.19%, respectively. The inclusion of the OESP as a spatial constraint of PEP, incorporating the limitation of ES space into the scenario setting, effectively mitigates the adverse impacts of the urbanization process on the Bay Area.

### 4.3 Landscape pattern index changes under different scenarios

**Fig 9:**
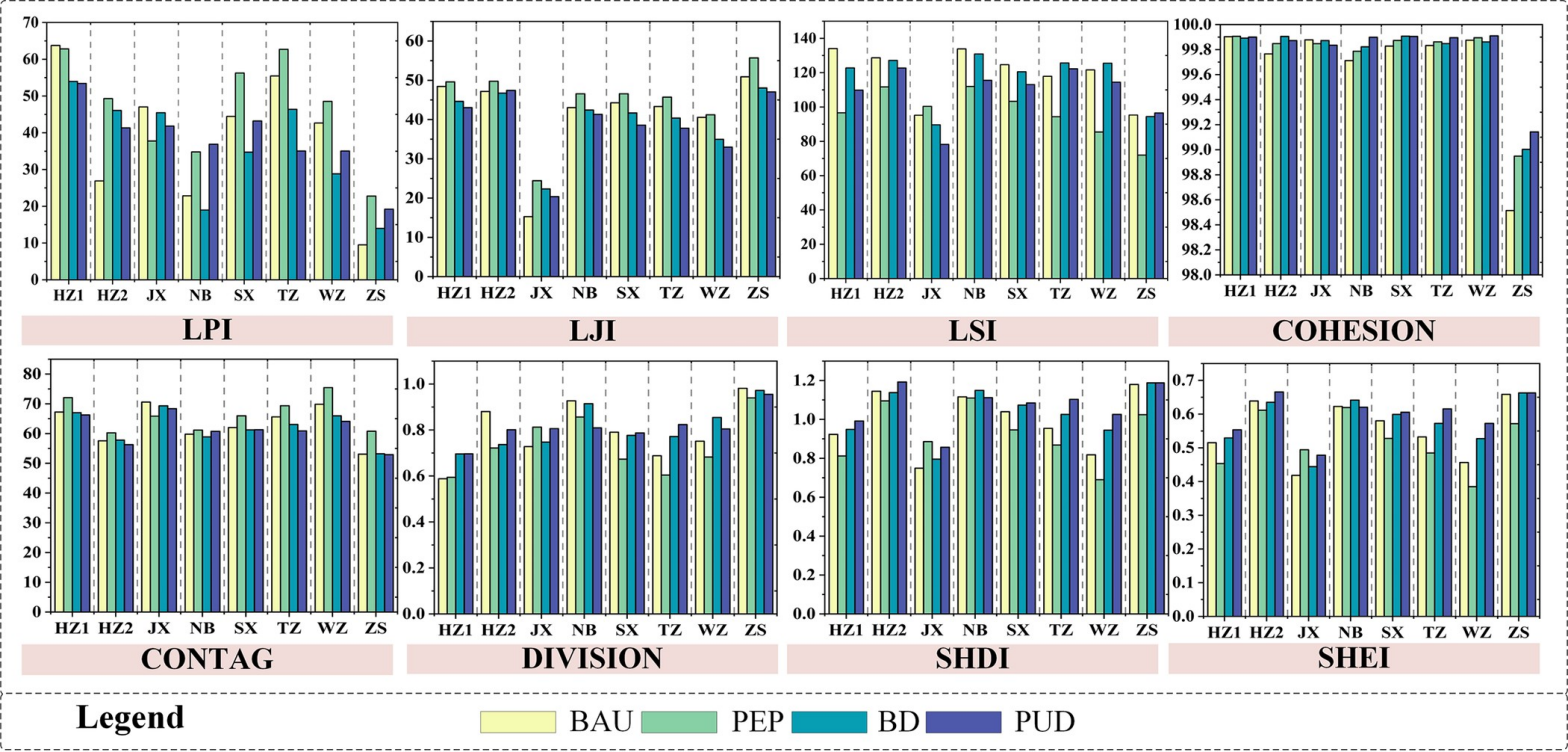
Landscape pattern indices in each city.

By calculating the landscape pattern index, we obtained the spatial structural characteristics of land use in each scenarios for each city, as shown in Fig 9. In the PEP scenario, the LPI, LJI, and CONTAG indices of each city reach the maximum values among the four scenarios, while the LSI, SHDI, and SHEI indices are generally at the minimum values among the four scenarios. In this scenario, the ZGBA exhibits increased patch aggregation, reduced fragmentation and diversity, and good landscape connectivity in each city. Under the constraint of the OESP, aggregation degree of each land use type increases, and the ESMP-SD-PLUS model succinctly controls the landscape fragmentation caused by the generation of small patches, enhancing the connectivity of large patches and achieving effective protection of the ecosystem. Under the constraint of the BESP, the landscape characteristics in the PUD scenario show a trend opposite to that of the PEP scenario. In the PUD scenario, the fragmentation and diversity of patches in the ZGBA increase, while connectivity and aggregation decrease. This is primarily on account of the extensive sprawl of construction land and cultivated land, which leads to increased fragmentation of large patches, as confirmed by the performance of the DIVISION, SHDI, and SHEI indices. The COHESION index values are relatively high and similar among all scenarios, indicating that the core areas of patches in each city are relatively continuous and large, and the connectivity of patches is relatively stable. Overall, the fragmentation degree of ZGBA is lower and the connectivity is better in the PEP and BAU scenarios. On the part of landscape diversity, most areas show the trend of PUD > BD > BAU > PEP in terms of SHDI and SHEI values, indicating a positive correlation between landscape heterogeneity and the economic development process. Regarding landscape aggregation, the changing trend of CONTAG and LJI generally follows PEP > BAU > BD > PUD. This indicates that under the ecological constraints of the optimal ESP, the transformation of patches in the ZGBA is concentrated in the boundary areas of patches and relatively dispersed. Additionally, the BAU scenario shows a landscape pattern balance and coordination second only to the PEP scenario. This is because the BAU scenario only considers the inertia of past LUCC without directly considering the impact of socio-economic elements such as the economy and population. In reality, it is extremely difficult to maintain the development speed of factors influencing LUCC unchanged, but the setting of the BAU scenario can serve as a reference benchmark for different development paths.

In general, the lower the intensity of ecological protection measures, the lower the aggregation and connectivity of landscape patches. Such aggregation and connectivity prevent the excessive occupation of ecological land by development, effectively protect ecological resources, and lead to higher differentiation levels among different land use, resulting in higher spatial heterogeneity. Therefore, by incorporating the ESP as a constraint for ecological protection space, in the quantitative simulation of the SD model and the spatial simulation of the PLUS model, significant differences exist in the landscape patterns and spatial characteristics among cities. The landscape aggregation and heterogeneity are higher under PEP and BAU than under BD and PUD, while the fragmentation and diversity are higher in the BD and PUD scenarios than in the PEP and BAU scenarios. The rapid economic development will be an important intrinsic driving force influencing the spatial fragmentation and aggregation in the ZGBA.

## 5 Discussion

### 5.1 Land use optimization significance of ESMP-SD-PLUS model

LUP is prime aiming to protect ecological land and functions to the maximum extent while meeting the needs of economic development and human activities [57]. Decreasing changes in ecological land shows an important requirement for optimizing land use and protecting ecological resources [58]. Many studies have combined LUCC with ESP to optimize regional eco-networks from a prediction perspective [27, 59] or apply Ecological Source-Sink Areas (ESSA) as spatial constraints [24]. In the process of LUCC simulation, the use of ecological space as a constraint has been widely applied in urban boundaries [19, 39, 60], and county-level development [17]. However, traditional ESP cannot reflect the impact of rapid urbanization development, lacks stability [8, 61], and has limited scenario-setting capabilities [17]. ESMP starts from the perspective of ES and forms a multi-level elastic constraint, which can effectively limit future LUCC in different scenarios. Currently, the spatiotemporal dynamic evolution of ESP and its impact on future LUCC have been extensively studied in the Pearl River Delta region [10, 14, 24, 62]. In addition, the SD model is considered to have the highest accuracy among quantitative models in bay area studies [10, 21], while the PLUS model will effectively overcome the shortcomings of previous LUCC simulation models [22]. Therefore, by coupling the quantitative predictive ability of the SD model with the spatial capability of the PLUS model, the ESMP-SD-PLUS model can effectively constrain the spatiotemporal evolution of LUCC. In our research, the results show that even under BAU without any ecological protection measures, the area of forest is still declining. The primary cause for such phenomenon is that the economic development of the bay area inevitably drives rapid growth in cultivated land and construction land, resulting in the loss of ecological resources such as forest and meadow. According to the strategic plan proposed by the Zhejiang Provincial Government in 2021 to "Construct the Zhejiang Greater Bay Area," the future development of the ZGBA will effectively promote urban integration. The prediction results of the PEP scenario validate the effective protection of ecological land by the ESMP-SD-PLUS model. By using ESMP as a multi-scenario spatial constraint, LUCC spatial changes become more compact, and under these constraints, high ecosystem service value of land resources are not encroached upon and even receive a certain degree of development. In summary, the ESMP-SD-PLUS model can minimize the negative effects of ZGBA development. The model can also furnish scientific references for the construction of similar bay areas and urban clusters.

### 5.2 Comparison of future development simulations in other bay area and similar areas

ZGBA, GBA and Bohai Greater Bay Area (BGBA) are the three major bay areas in China, sharing similar geographical conditions and spatial structural characteristics. However, there have been fewer studies on LUCC simulation in ZGBA and BGBA compared to the GBA. Therefore, comparing the future development paths and LUCC evolution of different bay areas will help promote coordinated development and adopt context-specific measures.

Contrasting with the GBA, in its future development process, the GBA (Pearl River Delta region) has experienced urban expansion and agglomeration in the estuarine areas [10]. With the advantageous geographical location and excellent deep-water ports at the estuary of the Pearl River, the GBA is one of the largest regional port clusters in the world [14]. Research indicates that future construction land in the GBA will mainly concentrate in the estuarine areas [8, 10, 14], and this trend is also reflected in the surrounding Fujian-Triangle region [28]. Under PUD, construction land in the bay area will rapidly increase, and the process of urban integration will become more evident [8, 10]. This is similar to the future development of ZGBA. ZGBA, relying on the estuary of the Qiantang River and the Ningbo-Zhoushan Port, shares similar bay area characteristics. Our results indicate that future urban construction land of ZGBA will surround the estuary and the Ningbo-Zhoushan Port, presenting an integrated development pattern. Therefore, ZGBA can learn from the development of the GBA and formulate land use planning accordingly.

Contrasting with the research area of the Hangzhou Bay, ZGBA has the Hangzhou Bay as its core development zone, promoting the coordinated development of the port industry belt in NB, TZ, and WZ, making it the most economically promising economic region in China. Research shows that although the increase in construction land in the Hangzhou Bay region is inevitable even under multi-objective optimization constraints [63], the ecological resources in the Hangzhou Bay area are limited, and the rapid economic development process generates a stress to the ES of the region. This is also supported by research on LUCC simulation in the Yangtze River Delta [24, 64]. Therefore, for the coordinated development of multiple cities in ZGBA, effective ecological resource reserves are needed for the future development of the Hangzhou Bay area.

Contrasting with existing research, the current ES of ZGBA shows a "lower in the north and higher in the south" distribution pattern, with the areas of insecurity concentrated in HZ1, JX, and NB [65]. Researchers such as Cui [18] simulated LUCC under different scenarios by modifying the Markov probability matrix, and their inertia scenario (S1), excessive expansion scenario (S2), and ecological protection scenario (S3) generally follow the same development strategies as BAU, PUD, and PEP in this study. Their results are similar to the findings of this study, with the focus areas of future urban expansion also being the Wenzhou Bay and Taizhou Bay. This indicates that future development in ZGBA should also pay attention to the Wenzhou Bay and Taizhou Bay.

### 5.3 Municipal land management strategy

PEP is the optimal scenario for holding the ecosystems of various cities in ZGBA. Forests are the main stronghold for protecting ecosystems. Under the PEP scenario, the forest area in each city increases rapidly, making it the best scenario for decreasing landscape fragmentation. In the southeast parts of NB and HZ, the PEP scenario effectively maintains the landscape aggregation of forests, resulting in higher spatial structural stability compared to the BD and PUD scenarios, and similar features are observed in TZ and WZ. Additionally, urban expansion is a key factor affecting landscape fragmentation. Urban expansion encroaches upon cultivated land and forests, more cultivated land needs to be developed. This is particularly evident in JX under the PUD scenario, and a similar feature is observed in the coastal areas of HZ1, WZ, and TZ. Considering the demand of high-quality economic development, the development plan under the BD scenario can be adopted for the Hangzhou Bay area (JX, HZ2, HZ1, SX, NB) to effectively alleviate the imbalance in LUCC structure caused by rapid urbanization [17]. TZ, WZ, and Fuyang County in HZ1, as the reserve resource areas for ecological forests in ZGBA, are crucial guarantees for maintaining the sustainable development of the bay area’s ecology. It is essential to reduce urbanization interference in these areas and choose the PEP or BAU scenario to maintain the ES. ZS possesses the richest fishery resources in the country and has a natural deep-water port. Its LUCC structure exhibits strong island attributes [66]. ZS mainly relies on fishing, aquaculture, port economy, and tourism, with a low environmental carrying capacity. The PEP scenario will be the best development scenario for ZS in the future, thus a better ecological environment will contribute to the development of tourism in ZS. In the context of rural revitalization, ecological development projects will help address the issues brought about by the coordinated development of the bay area’s cities [47].

Furthermore, the LUP planning of each city in the bay area is an important elements influencing future land use policies. Future LUCC should rely on development plans, policies, and regulations to prevent LUCC that deviate from development goals. For example, the land use development plan of Hangzhou City (2006–2020) aims to protect cultivated land and optimize the structure and layout of urban and rural land use, while the land use development plan of Hangzhou City (2021–2035) aims to anchor ecology, promote land intensification, and prevent disorderly urban sprawl. At the same time, it is necessary to establish intensive mechanisms for sustainable land use to prevent land degradation. Under the guidance of the "rural revitalization" strategy, efforts should be made to vigorously develop rural ecological tourism and reform land acquisition methods [67], promoting the integrated development of the "three-dimensional space (production space, living space, ecological space)".

### 5.4 Innovation, limitation and prosperity

On the basis of ESMP-SD-PLUS model, we predict the LUCC of ZGBA in a spatial-temporal perspective in 2035. By establishing an ESMP-based ecological protection spatial constraint, we set contrasting scenarios to provide reference for the development of ZGBA. This study applies the SD model to construct multi-scenario simulations that can characterize LUCC in the ZGBA from the perspectives of regional policies, social development, and natural conditions [13, 68, 69]. Furthermore, this model can be used to establish different types of spatial constraints for other research areas, such as water conservation protection in arid regions, conflicts between meadow and livestock in large-scale ranches, and sensitivity assessments in ecological restoration areas.

However, there are some limitations to our study. Firstly, the SSP data used to construct the PEP, BD, and PUD scenarios for future LUCC development are incomplete and subject to a certain degree of subjectivity. In further research, it would be beneficial to find more complete CMIP6 data and other parameters to establish more objective SSP scenarios [21]. Secondly, the spatial data used in the study have inconsistent resolutions. Resampling was performed to unify the data format to 30m, and interpolation methods were used to fill in missing values at some boundaries. These values may differ from the actual situation. In the future, spatial data with a resolution close to 30m should be selected for downscaled analysis. Thirdly, our study neglects the spatial connections brought by urban synergy and integration. Future research could utilize population migration data and indices of digital urban economic competitiveness to optimize the simulation of LUCC. Lastly, maintaining ES in the bay area requires both the integrity of terrestrial ecosystems and the inclusion of land-ocean interactions [8]. Therefore, in future simulation processes, further exploration is needed on the influence of marine ecosystems on LUCC in the bay area.

## 6 Conclusion

The ESMP-SD-PLUS model was proposed to simulate LUCC in a spatial-temporal perspective under the constraints of spatial limitations and quantitative requirements. The four ecosystem services in the bay area were evaluated, and ESMP constraints were development using the MSPA and MCR model. Based on the future development direction of the bay area, different scenarios were set up for comparison. The SD model and Markov model were selected to predict the land demand under the four scenarios in 2035. The ecological red line areas and ESMP were coupled and adjusted according to the development objectives of different scenarios.

The results indicate that the ESMP-SD-PLUS model can effectively simulate future LUCC in the bay area (Kappa = 0.92). Compared to traditional land use restrictions such as water and cultivated land, ESMP plays an important role in controlling land conversion and reducing ecological land degradation. By coupling the SD model for quantitative prediction and setting up scenario comparisons, it can provide path choices for the development of the ZGBA. Furthermore, the model can effectively reduce the negative changes caused by LUCC, and the overall landscape pattern and spatial characteristics under different scenarios are ranked as PEP > BAU > BD > PUD. Although PEP is the optimal development scenario in the ES protection perspective of the ZGBA, the development goals and paths of each city in the bay area under the integration process are not the same. Considering the need for economic development, Jiaxing, Huzhou, Hangzhou (except Fuyang County), Shaoxing, and Ningbo can choose BD as the future development scenario. Wenzhou, Taizhou, and Fuyang County in Hangzhou have abundant ecological resources, serving as the overall guarantee and reserve resources for the ES of the ZGBA, and are recommended to adopt the PEP scenario. Due to its unique island attributes and reliance on fisheries, port economy, and tourism industry, the Zhoushan area is recommended to adopt the PEP scenario for future development.

## Supporting information

S1 Appendix (DOCX)

## Abbreviation of professional terms

LUCC: Land Use and Land Cover Change
ZGBA: Zhejiang Greater Bay Area
SD: System Dynamics
PLUS: Patch-based Land Use Simulation model
ESP: Ecological Security Pattern
ESMP: Ecological Security Multi-scenario Pattern
LUP: Land Use Optimization Patterns
MCR: Minimum Cumulative Resistance Model
ES: Ecological Security
MSPA: Morphological Spatial Pattern Analysis
CS: Carbon Storage
SDR: Sediment Delivery Ratio
HQ: Habitat Quality
WY: Water Yield
BAU: Business As Usual
PEP: Priority of Ecological Protection
BD: Balanced Development
PUD: Priority of Urban development
GBA: Guangdong-Hong Kong-Macao Greater Bay Area

## References

[1] Wood S.L.R.; Jones S.K.; Johnson J.A.; Brauman K.A.; Chaplin-Kramer R.; Fremier A.; et al. Distilling the role of ecosystem services in the Sustainable Development Goals. *Ecosystem Services* 2018, 29, 70–82, doi: 10.1016/j.ecoser.2017.10.010

[2] Sun L.; Chen J.; Li Q.; Huang D. Dramatic uneven urbanization of large cities throughout the world in recent decades. *Nat Commun* 2020, 11, 5366, doi: 10.1038/s41467-020-19158-1 33097712 PMC7584620

[3] Newbold T.; Hudson L.N.; Hill S.L.; Contu S.; Lysenko I.; Senior R.A.; et al. Global effects of land use on local terrestrial biodiversity. *Nature* 2015, 520, 45–50, doi: 10.1038/nature14324 25832402

[4] Zhao S.; Da L.; Tang Z.; Fang H.; Song K.; Fang J. Ecological consequences of rapid urban expansion: Shanghai, China. *Frontiers in Ecology and the Environment* 2006, 4, 341–346, doi: 10.1890/1540-9295(2006)004[0341:Ecorue]2.0.Co;2

[5] Feng Y.; Lei Z.; Tong X.; Gao C.; Chen S.; Wang J.; et al. Spatially-explicit modeling and intensity analysis of China’s land use change 2000–2050. *J Environ Manage* 2020, 263, 110407, doi: 10.1016/j.jenvman.2020.110407 32174538

[6] Butchart S.H.; Walpole M.; Collen B.; van Strien A.; Scharlemann J.P.; Almond R.E.; et al. Global biodiversity: indicators of recent declines. *Science* 2010, 328, 1164–1168, doi: 10.1126/science.1187512 20430971

[7] Lai L.; Huang X.; Yang H.; Chuai X.; Zhang M.; Zhong T.; et al. Carbon emissions from land-use change and management in China between 1990 and 2010. *Sci Adv* 2016, 2, e1601063, doi: 10.1126/sciadv.1601063 27847866 PMC5099982

[8] Li L.; Huang X.; Wu D.; Yang H. Construction of ecological security pattern adapting to future land use change in Pearl River Delta, China. *Applied Geography* 2023, 154, doi: 10.1016/j.apgeog.2023.102946

[9] Liang X.; Liu X.; Li D.; Zhao H.; Chen G. Urban growth simulation by incorporating planning policies into a CA-based future land-use simulation model. *International Journal of Geographical Information Science* 2018, 32, 2294–2316, doi: 10.1080/13658816.2018.1502441

[10] Jiao M.; Hu M.; Xia B. Spatiotemporal dynamic simulation of land-use and landscape-pattern in the Pearl River Delta, China. *Sustainable Cities and Society* 2019, 49, doi: 10.1016/j.scs.2019.101581

[11] Liu X.; Liang X.; Li X.; Xu X.; Ou J.; Chen Y.; et al. A future land use simulation model (FLUS) for simulating multiple land use scenarios by coupling human and natural effects. *Landscape and Urban Planning* 2017, 168, 94–116, doi: 10.1016/j.landurbplan.2017.09.019

[12] Estacio I.; Sianipar C.P.M.; Onitsuka K.; Basu M.; Hoshino S. A statistical model of land use/cover change integrating logistic and linear models: An application to agricultural abandonment. *International Journal of Applied Earth Observation and Geoinformation* 2023, 120, doi: 10.1016/j.jag.2023.103339

[13] Verburg P.H.; Soepboer W.; Veldkamp A.; Limpiada R.; Espaldon V.; Mastura S.S. Modeling the spatial dynamics of regional land use: the CLUE-S model. *Environ Manage* 2002, 30, 391–405, doi: 10.1007/s00267-002-2630-x 12148073

[14] Li Z.-T.; Li M.; Xia B.-C. Spatio-temporal dynamics of ecological security pattern of the Pearl River Delta urban agglomeration based on LUCC simulation. *Ecological Indicators* 2020, 114, doi: 10.1016/j.ecolind.2020.106319

[15] Zhang Z.; Hu B.; Jiang W.; Qiu H. Identification and scenario prediction of degree of wetland damage in Guangxi based on the CA-Markov model. *Ecological Indicators* 2021, 127, doi: 10.1016/j.ecolind.2021.107764

[16] Zhou L.; Dang X.; Sun Q.; Wang S. Multi-scenario simulation of urban land change in Shanghai by random forest and CA-Markov model. *Sustainable Cities and Society* 2020, 55, doi: 10.1016/j.scs.2020.102045

[17] Nie W.; Xu B.; Yang F.; Shi Y.; Liu B.; Wu R.; et al. Simulating future land use by coupling ecological security patterns and multiple scenarios. *Sci Total Environ* 2023, 859, 160262, doi: 10.1016/j.scitotenv.2022.160262 36400298

[18] Cui W.; Cai L.; Xi H.; Yang F.; Chen M. Ecological Security assessment and multi-scenario simulation analusis of Zhejiang Greater Bay Area Based on LUCC. *Acta Ecologica Sinica* 2022, 42, 2136–2148.

[19] Yao Z.; Jiang C.; Shan-shan F. Effects of urban growth boundaries on urban spatial structural and ecological functional optimization in the Jining Metropolitan Area, China. *Land Use Policy* 2022, 117, doi: 10.1016/j.landusepol.2022.106113

[20] Wang Z.; Li X.; Mao Y.; Li L.; Wang X.; Lin Q. Dynamic simulation of land use change and assessment of carbon storage based on climate change scenarios at the city level: A case study of Bortala, China. *Ecological Indicators* 2022, 134, doi: 10.1016/j.ecolind.2021.108499

[21] Wu J.; Luo J.; Zhang H.; Qin S.; Yu M. Projections of land use change and habitat quality assessment by coupling climate change and development patterns. *Sci Total Environ* 2022, 847, 157491, doi: 10.1016/j.scitotenv.2022.157491 35870584

[22] Liang X.; Guan Q.; Clarke K.C.; Liu S.; Wang B.; Yao Y. Understanding the drivers of sustainable land expansion using a patch-generating land use simulation (PLUS) model: A case study in Wuhan, China. *Computers*, *Environment and Urban Systems* 2021, 85, doi: 10.1016/j.compenvurbsys.2020.101569

[23] Liao G.; He P.; Gao X.; Lin Z.; Huang C.; Zhou W.; et al. Land use optimization of rural production–living–ecological space at different scales based on the BP–ANN and CLUE–S models. *Ecological Indicators* 2022, 137, doi: 10.1016/j.ecolind.2022.108710

[24] Zhang D.; Wang X.; Qu L.; Li S.; Lin Y.; Yao R.; et al. Land use/cover predictions incorporating ecological security for the Yangtze River Delta region, China. *Ecological Indicators* 2020, 119, doi: 10.1016/j.ecolind.2020.106841

[25] Liang X.; Liu X.; Li X.; Chen Y.; Tian H.; Yao Y. Delineating multi-scenario urban growth boundaries with a CA-based FLUS model and morphological method. *Landscape and Urban Planning* 2018, 177, 47–63, doi: 10.1016/j.landurbplan.2018.04.016

[26] Liang J.; He X.; Zeng G.; Zhong M.; Gao X.; Li X.; et al. Integrating priority areas and ecological corridors into national network for conservation planning in China. *Sci Total Environ* 2018, 626, 22–29, doi: 10.1016/j.scitotenv.2018.01.086 29331835

[27] Li Y.; Liu W.; Feng Q.; Zhu M.; Yang L.; Zhang J.; et al. The role of land use change in affecting ecosystem services and the ecological security pattern of the Hexi Regions, Northwest China. *Sci Total Environ* 2023, 855, 158940, doi: 10.1016/j.scitotenv.2022.158940 36152856

[28] Liu X.; Wei M.; Li Z.; Zeng J. Multi-scenario simulation of urban growth boundaries with an ESP-FLUS model: A case study of the Min Delta region, China. *Ecological Indicators* 2022, 135, doi: 10.1016/j.ecolind.2022.108538

[29] Li S.; Xiao W.; Zhao Y.; Lv X. Incorporating ecological risk index in the multi-process MCRE model to optimize the ecological security pattern in a semi-arid area with intensive coal mining: A case study in northern China. *Journal of Cleaner Production* 2020, 247, doi: 10.1016/j.jclepro.2019.119143

[30] Peng J.; Li H.; Liu Y.; Hu Y.n.; Yang Y. Identification and optimization of ecological security pattern in Xiong’an New Area. *Acta Geographica Sinica* 2018, 73, 701–710.

[31] McDonald R.I. Global urbanization: can ecologists identify a sustainable way forward? *Frontiers in Ecology and the Environment* 2008, 6, 99–104, doi: 10.1890/070038

[32] Perkl R.M. Geodesigning landscape linkages: Coupling GIS with wildlife corridor design in conservation planning. *Landscape and Urban Planning* 2016, 156, 44–58, doi: 10.1016/j.landurbplan.2016.05.016

[33] Raudsepp-Hearne C.; Peterson G.D.; Bennett E.M. Ecosystem service bundles for analyzing tradeoffs in diverse landscapes. *Proc Natl Acad Sci U S A* 2010, 107, 5242–5247, doi: 10.1073/pnas.0907284107 20194739 PMC2841950

[34] Verburg P.H.; Chen Y. Multiscale Characterization of Land-Use Patterns in China. *Ecosystems* 2000, 3, 369–385, doi: 10.1007/s100210000033

[35] Yang H.; Huang X.; Thompson J.R.; Flower R.J. China’s soil pollution: urban brownfields. *Science* 2014, 344, 691–692, doi: 10.1126/science.344.6185.691-b 24833374

[36] Qiu L.; Pan Y.; Zhu J.; Amable G.S.; Xu B. Integrated analysis of urbanization-triggered land use change trajectory and implications for ecological land management: A case study in Fuyang, China. *Sci Total Environ* 2019, 660, 209–217, doi: 10.1016/j.scitotenv.2018.12.320 30640089

[37] Jiang B.; Bai Y.; Wong C.P.; Xu X.; Alatalo J.M. China’s ecological civilization program–Implementing ecological redline policy. *Land Use Policy* 2019, 81, 111–114, doi: 10.1016/j.landusepol.2018.10.031

[38] Tan Z.; Guan Q.; Lin J.; Yang L.; Luo H.; Ma Y.; et al. The response and simulation of ecosystem services value to land use/land cover in an oasis, Northwest China. *Ecological Indicators* 2020, 118, doi: 10.1016/j.ecolind.2020.106711

[39] Ma S.; Wang L.-J.; Zhu D.; Zhang J. Spatiotemporal changes in ecosystem services in the conservation priorities of the southern hill and mountain belt, China. *Ecological Indicators* 2021, 122, doi: 10.1016/j.ecolind.2020.107225

[40] Jiang T.; Su B.; Wang Y. Gridded datasets for population and economy under Shared Socioeconomic Pathways. 2022, 10.57760/sciencedb.01683.

[41] Yang J.; Huang X. The 30 m annual land cover dataset and its dynamics in China from 1990 to 2019. *Earth System Science Data* 2021, 13, 3907–3925, doi: 10.5194/essd-13-3907-2021

[42] Zhao N.; Liu Y.; Cao G.; Samson E.L.; Zhang J. Forecasting China’s GDP at the pixel level using nighttime lights time series and population images. *GIScience & Remote Sensing* 2017, 54, 407–425, doi: 10.1080/15481603.2016.1276705

[43] Yue T.; Yin S.; Xie Y.; Yu B.; Liu B. Rainfall erosivity mapping over mainland China based on high-density hourly rainfall records. *Earth System Science Data* 2022, 14, 665–682, doi: 10.5194/essd-14-665-2022

[44] Peng S.; Ding Y.; Wen Z.; Chen Y.; Cao Y.; Ren J. Spatiotemporal change and trend analysis of potential evapotranspiration over the Loess Plateau of China during 2011–2100. *Agricultural and Forest Meteorology* 2017, 233, 183–194, doi: 10.1016/j.agrformet.2016.11.129

[45] Ding Y.; Wang L.; Gui F.; Zhao S.; Zhu W. Ecosystem Carbon Storage in Hangzhou Bay Area Based on InVEST and PLUS Models. *Environmental Science* 2022, 44, 3343–3352. 10.13227/j.hjkx.202204080 37309952

[46] Fu Y.; Shi X.; He J.; Yuan Y.; Qu L. Identification and optimization strategy of county ecological security pattern: A case study in the Loess Plateau, China. *Ecological Indicators* 2020, 112, doi: 10.1016/j.ecolind.2019.106030

[47] Nie W.; Shi Y.; Siaw M.J.; Yang F.; Wu R.; Wu X.; et al. Constructing and optimizing ecological network at county and town Scale: The case of Anji County, China. *Ecological Indicators* 2021, 132, doi: 10.1016/j.ecolind.2021.108294

[48] Posner S.; Verutes G.; Koh I.; Denu D.; Ricketts T. Global use of ecosystem service models. *Ecosystem Services* 2016, 17, 131–141, doi: 10.1016/j.ecoser.2015.12.003

[49] Hall L.S.; Krausman P.R.; Morrison M.L. The habitat concept and a plea for standard terminology. *Wildlife Society Bulletin* 1997, 25, 173–182.

[50] Petrosillo I.; Zaccarelli N.; Semeraro T.; Zurlini G. The effectiveness of different conservation policies on the security of natural capital. *Landscape and Urban Planning* 2009, 89, 49–56, doi: 10.1016/j.landurbplan.2008.10.003

[51] Sharp R.; Tallis H.T.; Ricketts T.; Guerry A.D.; Wood S.A.; ChaplinKramer R.; et al. InVEST +VERSION+ User’s Guide. *The natural capital project*, Stanford University 2016.

[52] Adriaensen F.; Chardon J.P.; De Blust G.; Swinnen E.; Villalba S.; Gulinck H.; et al. The application of ‘least-cost’ modelling as a functional landscape model. *Landscape and Urban Planning* 2003, 64, 233–247, doi: 10.1016/s0169-2046(02)00242-6

[53] Santos J.S.; Leite C.C.C.; Viana J.C.C.; dos Santos A.R.; Fernandes M.M.; de Souza Abreu V.; et al. Delimitation of ecological corridors in the Brazilian Atlantic Forest. *Ecological Indicators* 2018, 88, 414–424, doi: 10.1016/j.ecolind.2018.01.011

[54] China, G.o.P.s.R.o. Zhejiang Greater Bay Area Construction Plan. Available online: (accessed on 07–15).

[55] Pontius R.G.; Millones M. Death to Kappa: birth of quantity disagreement and allocation disagreement for accuracy assessment. *International Journal of Remote Sensing* 2011, 32, 4407–4429, doi: 10.1080/01431161.2011.552923

[56] Bao S.; Yang F. Spatio-Temporal Dynamic of the Land Use/Cover Change and Scenario Simulation in the Southeast Coastal Shelterbelt System Construction Project Region of China. *Sustainability* 2022, 14, doi: 10.3390/su14148952

[57] Zheng W.; Ke X.; Xiao B.; Zhou T. Optimising land use allocation to balance ecosystem services and economic benefits—A case study in Wuhan, China. *J Environ Manage* 2019, 248, 109306, doi: 10.1016/j.jenvman.2019.109306 31466175

[58] Wang W.; Jiao L.; Jia Q.; Liu J.; Mao W.; Xu Z.; et al. *Land use optimization modelling with ecological priority perspective for large-scale spatial planning*. *Sustainable Cities and Society* 2021, 65, doi: 10.1016/j.scs.2020.102575

[59] Yang Liangjie; Wang J.; Wei W.; Yang Y.; Guo Z. Ecological security pattern construction and optimization in Arid Inland River Basin: A case study of Shiyang River Basin. *Acta Ecologica Sinica* 2020, 40, 5915–5927, doi: 10.5846/stxb201908281782

[60] Li Y.; Ma Q.; Song Y.; Han H. Bringing conservation priorities into urban growth simulation: An integrated model and applied case study of Hangzhou, China. *Resources*, *Conservation and Recycling* 2019, 140, 324–337, doi: 10.1016/j.resconrec.2018.09.032

[61] Hou W.; Zhou W.; Li J.; Li C. Simulation of the potential impact of urban expansion on regional ecological corridors: A case study of Taiyuan, China. *Sustainable Cities and Society* 2022, 83, doi: 10.1016/j.scs.2022.103933

[62] Jiang H.; Peng J.; Dong J.; Zhang Z.; Xu Z.; Meersmans J. Linking ecological background and demand to identify ecological security patterns across the Guangdong-Hong Kong-Macao Greater Bay Area in China. *Landscape Ecology* 2021, 36, 2135–2150, doi: 10.1007/s10980-021-01234-6

[63] Zhang T.; Xin X.; He F.; Wang X.; Chen K. How to promote sustainable land use in Hangzhou Bay, China? A decision framework based on fuzzy multiobjective optimization and spatial simulation. *Journal of Cleaner Production* 2023, 414, doi: 10.1016/j.jclepro.2023.137576

[64] Zhang Z.; Wang X.; Zhang Y.; Gao Y.; Liu Y.; Sun X.; et al. Simulating land use change for sustainable land management in rapid urbanization regions: a case study of the Yangtze River Delta region. *Landscape Ecology* 2023, 38, 1807–1830, doi: 10.1007/s10980-023-01657-3

[65] Cui W.; Chen M.; Zhong H. Spatial differentiation and differentiated management of ecological security in the Bay Area based on ESDA and GA: A case study of the Zhejiang Greater Bay Area. *Acta Ecologica Sinica* 2023, 43, 2074–2087.

[66] Cui W.; Xi H.; Cai l.; Chen M.; Xu C. Spatial and Temporal Change of Ecosystem Service Value in China’s Island Counties Base on NDVI. *Economic Geography* 2021, 41, 184–192.

[67] Gao J.; Liu Y.; Chen J. China’s initiatives towards rural land system reform. *Land Use Policy* 2020, 94, doi: 10.1016/j.landusepol.2020.104567

[68] Zhang D.; Huang Q.; He C.; Wu J. Impacts of urban expansion on ecosystem services in the Beijing-Tianjin-Hebei urban agglomeration, China: A scenario analysis based on the Shared Socioeconomic Pathways. *Resources*, *Conservation and Recycling* 2017, 125, 115–130, doi: 10.1016/j.resconrec.2017.06.003

[69] Li C.; Wu Y.; Gao B.; Zheng K.; Wu Y.; Li C. Multi-scenario simulation of ecosystem service value for optimization of land use in the Sichuan-Yunnan ecological barrier, China. *Ecological Indicators* 2021, 132, doi: 10.1016/j.ecolind.2021.108328

