# Supplementary material for: Future land use prediction and optimization strategy of Zhejiang Greater Bay Area coupled with ecological security multi-scenario pattern
Source: PLoS One. 2024 Apr 18;19(4):e0291570. doi: 10.1371/journal.pone.0291570 (PMC11025748; doi:10.1371/journal.pone.0291570)
Supplement: S1 Appendix — (DOCX) [file pone.0291570.s001.docx]

**Appendix**

**S1 Parameters of Future Development Scenario**

With reference to Jiao's research on Bay Area (Jiao et al., 2019) and the actual situation of ZGBA, parameters of SD model under different scenarios are set, as shown in Table S1.

Table S1 Parameters Setting under Different Development Scenarios

| variable | PEP | BD | PUD |
| --- | --- | --- | --- |
| GDP | SSP1 | SSP2 | SSP5 |
| Population | SSP1 | SSP2 | SSP5 |
| Invest ratio in primary industry in bay area | 0.02 | 0.01 | 0.005 |
| Agirculture output value ratio | 0.3 | 0.45 | 0.6 |
| Forestry output value ratio | 0.01 | 0.02 | 0.03 |
| Animal husbandary output value ratio | 0.15 | 0.125 | 0.01 |
| Crop sown area in bay area | 1200 | 1800 | 2500 |
| precipitation | 192890 | 17500 | 150000 |
| Effective irrigation area of cultivated land in bay area | 1200 | 1350 | 1500 |
| Quantity of pesticide usage | 26000 | 36000 | 46226 |
| Construction enterprise production in bay area | 10075.4 | 15000 | 20000 |
| Number of scientific patents in bay area | 420000 | 43500 | 450000 |
| Number of scientific research institutions in bay area | 80 | 90 | 100 |
| adjustment coefficient of construction land | 0.6 | 0.64 | 0.68 |

**S2 The assessment of ecosystem services value**

**S2.1 Carbon Storage**

The carbon storage of an ecosystem can be divided into four components: aboveground and belowground biomass carbon pools, soil carbon pool, and dead organic carbon pool (Ding et al., 2022), as shown in the Table S4.

Table S4 Carbon Pool

| LUCC | C_above | C_below | C_soil | C_dead |
| --- | --- | --- | --- | --- |
| Cultivated land | 18.9 | 12.5 | 85.5 | 2.4 |
| Forest | 36.3 | 7.3 | 125.8 | 3.4 |
| Meadow | 17.4 | 20.8 | 112.2 | 2.9 |
| Water | 0 | 0 | 81.1 | 0 |
| Construction land | 0 | 0 | 0 | 0 |
| Unused land | 24.3 | 4.9 | 74.6 | 2.2 |

**S2.2 Habitata Quality**

The threat factors of different land use types in the study area were set by reference to relevant literature (Nie et al., 2023; Zhu et al., 2020) and the InVEST model user manual (Sharp et al., 2016). The habitat quality sensitivity table is shown in Table S5 and the threat factor attenuation table is shown in Table S6. The formula for calculating habitat quality is as follows:

$$\begin{aligned} \text{Q}_{\text{xj}}\text{=}\text{H}_{\text{j}}\left( \text{1-}\left( \frac{\text{D}_{\text{xj}}^{\text{z}}}{\text{D}_{\text{xj}}^{\text{z}}\text{+}\text{k}^{\text{z}}} \right) \right)\#\left( 1 \right) \end{aligned}$$

Where, $\text{Q}_{\text{xj}}$ is the habitat quality of grid x of land use type j; $\text{H}_{\text{j}}$ is the habitat suitability of land use type j; $D_{\text{xj}}$ is the threat level of land use type grid x divided by threat factors. K is the scale constant; Z is the normalized constant.

Table S5 Sensitivity

| LUCC | Habitat | Cultivated land | Unused land | Construction land | Distance to national road | Distance to highway | Distance to province road | Distance to railway |
| --- | --- | --- | --- | --- | --- | --- | --- | --- |
| Cultivated land | 1 | 0.3 | 0.65 | 0.5 | 0.4 | 0.3 | 0.2 | 0.1 |
| Forest | 1 | 0.6 | 0.65 | 0.8 | 0.7 | 0.6 | 0.5 | 0.4 |
| Meadow | 1 | 0.3 | 0.35 | 0.5 | 0.4 | 0.3 | 0.2 | 0.1 |
| Water | 1 | 0.7 | 0.35 | 0.7 | 0.6 | 0.5 | 0.4 | 0.3 |
| Construction land | 0 | 0 | 0 | 0 | 0 | 0 | 0 | 1 |
| Unused land | 1 | 0.3 | 0.35 | 0.5 | 0.4 | 0.3 | 0.2 | 0.1 |

Threat factors can be divided into linear attenuation and exponential attenuation. The formualation is shown as follows:

linear attenuation：

$$\begin{aligned} \text{i}_{\text{rxy}}\text{=1- (}\frac{\text{d}_{\text{xy}}}{\text{d}_{\text{r max}}}\text{)}\text{ }\#\left( 2 \right) \end{aligned}$$

exponential attenuation：

$$\begin{aligned} \text{i}_{\text{rxy}}\text{= exp (- (}\frac{\text{2.99}}{\text{d}_{\text{r max}}}\text{) }\text{d}_{\text{xy}}\text{)} \#\left( 3 \right) \end{aligned}$$

where，$\text{i}_{\text{rxy}}$ is the pressure degree of threat factor on x; $\text{d}_{\text{xy}}$ is the distance between x and y; $\text{d}_{\text{r max}}$ is the biggest threat factor affecting distance.

Table S6 Threat

| THREAT | MAX_DIST | WEIGHT | DECAY |
| --- | --- | --- | --- |
| Cultivated land | 8 | 0.7 | Linear |
| Unused land | 5 | 0.6 | Linear |
| Construction land | 10 | 1 | Linear |
| Distance to national road | 2 | 0.6 | Exponential |
| Distance to highway | 3 | 0.8 | Exponential |
| Distance to province road | 1 | 0.4 | Exponential |
| Distance to railway | 4 | 1 | Exponential |

**S2.3 Water Yield**

The water yeild module of InVEST model is based on the principle of water balance, that is, the amount of water after precipitation minus the actual evapotranspiration is the water conservation amount. The ecological sensitivity table is shown in Table S7. The main formula of the model is as follows:

$$\begin{aligned} \text{Y (x) =(1-}\frac{\mathrm{AET}\left( x \right)}{P\left( x \right)}\text{) ×P (x)}\#\left( 4 \right) \end{aligned}$$

where，

$$\frac{\mathrm{AET}\left( x \right)}{P\left( x \right)}\text{ = 1+}\frac{\mathrm{PET}\left( x \right)}{P\left( x \right)}-[{\left( \frac{\mathrm{PET}\left( x \right)}{P\left( x \right)} \right)^{\omega\left( x \right)}\text{]}}^{\frac{1}{\omega\left( x \right)}} (5)$$

$$\begin{aligned} \omega\left( x \right)=Z\frac{\mathrm{AWC}\left( x \right)}{P\left( x \right)}+1.25\#\left( 6 \right) \end{aligned}$$

$$\mathrm{AWC}\left( x \right)=54.509-0.132 \times P_{\mathrm{SAN}}\text{ -0.003× (}{P_{\mathrm{SAN}})}^{2}\text{-0.055 × }P_{\mathrm{SIL}}\text{ -0.006} \text{× (}{P_{\mathrm{SIL}})}^{2}-0.738 \text{×}P_{\mathrm{CLA}}\text{ + 0.007} \text{× (}{P_{\mathrm{CLA}})}^{2}-2.688 \text{× }P_{C}+0.501 \times({P_{C})}^{2} (7)$$

Where,$\text{ }\text{Y (x)}$ is the water yield; $\text{P (x)}$ is the annual precipitation (mm); $AET(x)$ is the actual evapotranspiration. PET data was obtained from the TPDC (Peng et al., 2017).

$\mathrm{AWC}\left( x \right)$ is the water effectively used by vegetation; $P_{\mathrm{SAN}}$, $P_{\mathrm{SIL}}$, $P_{\mathrm{CLA}}$, $P_{C}$ are the contents of sand, powder, clay and organic carbon in soil, respectively; Zhang's constant reflects the total amount of water stored and released by plants from the soil (Zhang et al., 2001).

TableS7 Biophysical Table of WY

| LUCC | KC | Root_depth | LULC_veg |
| --- | --- | --- | --- |
| Cultivated land | 1.1 | 1000 | 1 |
| Forest | 1.008 | 3500 | 1 |
| Meadow | 0.865 | 2000 | 1 |
| Water | 1.05 | 10 | 0 |
| Construction land | 0.2 | 0 | 0 |
| Unused land | 0.15 | 500 | 0 |

**S2.4 Soil Retention**

The modified General Soil Erosion Equation (RUSLE) is used to evaluate soil conservation services in the ZGBA.

$$\begin{aligned} SDR=R\times K\times\mathrm{LS}\times\left( 1-C\times P \right)\#\left( 8 \right) \end{aligned}$$

Where, R is the rainfall erosivity factor, K is the soil erodibility factor, and LS is the slope length and slope factor, where L represents the slope length factor and S represents the slope factor, which are two factors reflecting the influence of terrain on soil erosion. C stands for vegetation cover and crop management factors; P stands for anthropogenic soil and water conservation factor.

The R value comes from the research results of Beijing Normal University (Yue et al., 2022). The K value is calculated using the HWSD database and the empirical formula (China, 2021) published by the Ministry of Ecology and Environment in August 2021, as follows:

$$\begin{aligned} K=\left( -0.01383+0.51575\times K_{\mathrm{epic}} \right)\times0.1317\#\left( 9 \right) \end{aligned}$$

$$\begin{aligned} K_{\mathrm{epic}}=\left( 0.2+0.3e^{-0.0256m_{s}\left( 1-\frac{m_{\mathrm{silt}}}{100} \right)} \right) \\ \times\left[ \frac{m_{\mathrm{silt}}}{m_{c}+m_{\mathrm{silt}}} \right]^{0.3}\times\left\{ 1-\frac{0.25orgC}{\left[ orgC+e^{\left( 3.72-2.95orgC \right)} \right]} \right\}\times\\ \left\{ 1-0.7\times\frac{1-\frac{m_{s}}{100}}{\left\{ \left( 1-\frac{m_{s}}{100} \right)+e^{\left[ 5.51+22.9\left( 1-\frac{m_{s}}{100} \right) \right]} \right\}} \right\}\#\left( 10 \right) \end{aligned}$$

Where, $K_{\mathrm{epic}}$represents soil erodibility factor before modification. K represents soil erodibility factor after modification. $m_{c}$,$m_{\mathrm{silt}}$,$m_{s}$, $\mathrm{orgC}$ and are clay particles (<0.002mm), silt particles (0.002mm ~ 0.05mm), sand particles (0.05mm ~ 2mm) and percentage content (%) of organic carbon, respectively.

The LS factor is calculated from DEM data. The values of C and P were set by reference to relevant literature(Cai et al., 2000; Liu et al., 2009; Nie et al., 2023). The Biophysical table is shown in Table S8.

TableS8 Biophysical Table of SDR

| LUCC | Usle_c | Usle_p |
| --- | --- | --- |
| Cultivated land | 0.5 | 0.5 |
| Forest | 0.3 | 1 |
| Meadow | 0.4 | 0.7 |
| Water | 0.4 | 0.6 |
| Construction land | 1 | 0 |
| Unused land | 0.6 | 0.3 |

**S3 Spatial Principal Component Analysis Processing**

Spatial Principal Component Analysis (SPCA), based on statistical principles and GIS, corresponds to a matrix for each spatial variable, and assigns the degree of influence of relevant spatial variables on dependent variables to corresponding principal component factors(Zerrouqi et al., 2008), which can eliminate information redundancy caused by possible correlations among factors (Zou and Yoshino, 2017). The specific formula is as follows:

$$\begin{aligned} RV=\sum_{k=1}^{m} a_{\mathrm{ik}}w_{k}= \sum_{k=1}^{m} \frac{a_{\mathrm{ij}}F_{k}}{\sum_{p=1}^{m} F_{k}}\#\left( 12 \right) \end{aligned}$$

$a_{\mathrm{ij}}$ is the i-th a grid of the k-th factor; $w_{j}$ and m are weight allocation and number of factors, respectively. P is the number of principal components; $F_{j}$ is the variance of the common factor.

Use ArcGIS 10.8 software for SPCA operations. The results are shown in Figure S3:


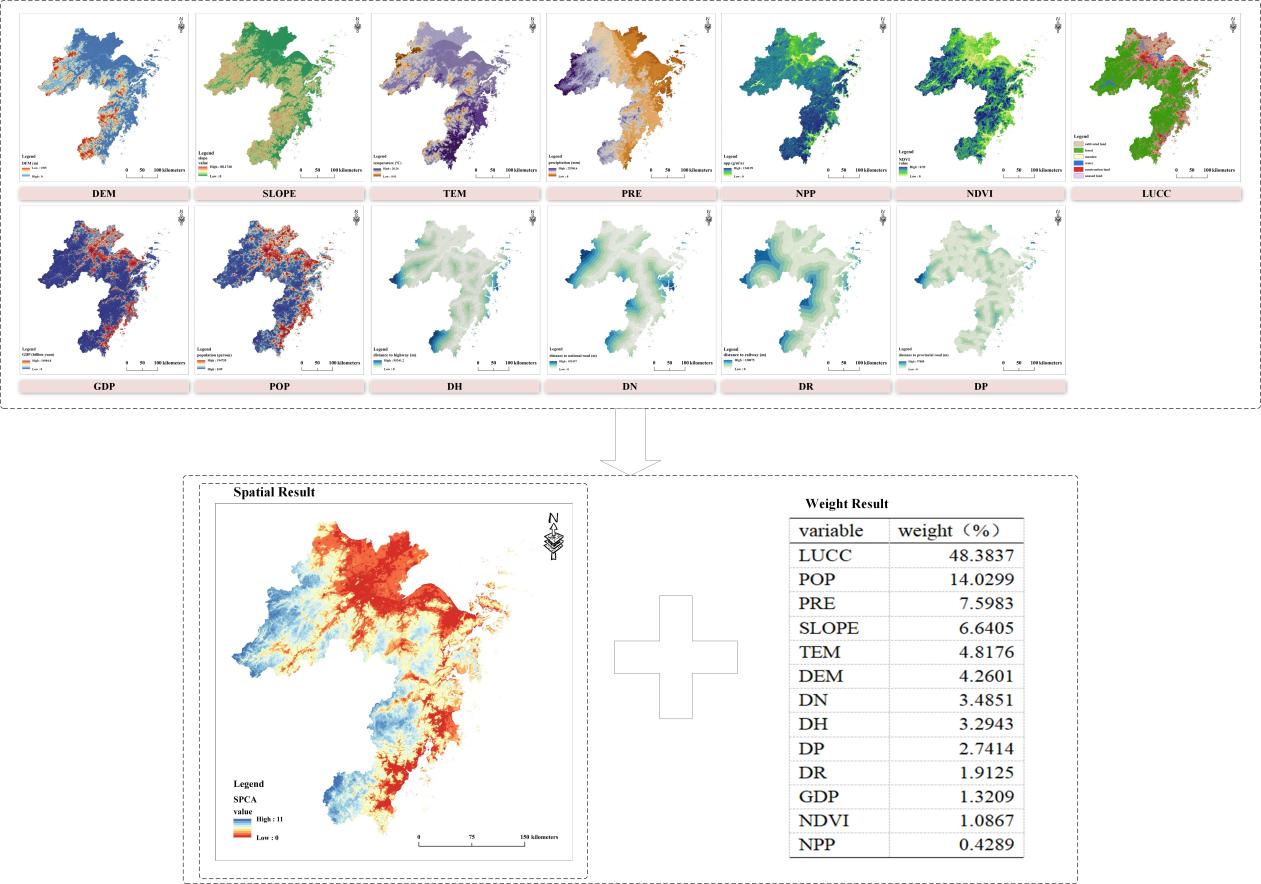


Figure S3 The Flowchat of SPCA Model

**S4 Minimum Cumulative Resistance Model**

MCR model is often used in the studies of species migration and landscape spatial planning, and it is the minimum cost for species to overcome spatial resistance from a certain ecological source to a destination(Bao and Yang, 2022a). The formula is as follows：

$$\begin{aligned} MCR=f_{\min}\sum_{j=n}^{i=m} D_{\mathrm{ij}}R_{i}\#\left( 11 \right) \end{aligned}$$

MCR is the minimum cumulative resistance diffusing from source j to any point in space. f is a function of the proportional relationship; $D_{\mathrm{ij}}$ is the distance between source j and any point in space through source i; $R_{i}$ is the spatial resistance value of source i.

**S5 Parameters of PLUS**

**S5.1 Selection of Driving Factors**

The driving factors selected in this paper are shown in Figure S2.


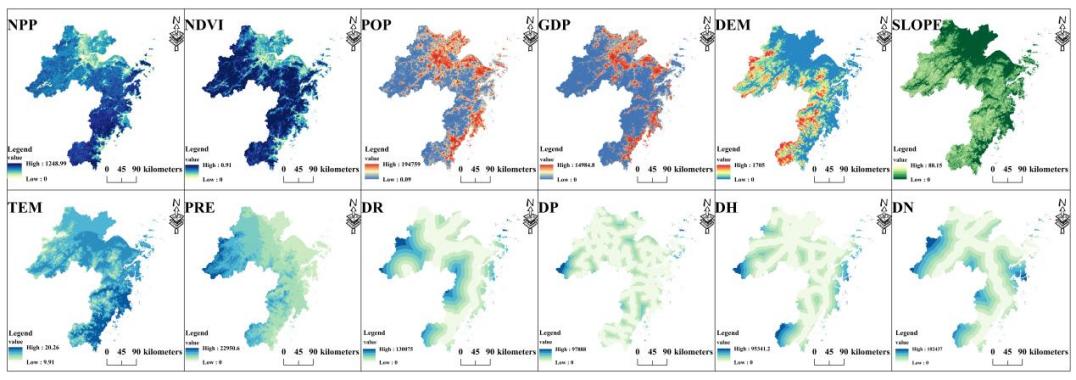


Figure S2 The Selection of Driving Factors

**S5.2 Parameters setting**

The future LUCC transfer matrix and neighborhood weight refer to the LUCC transfer matrix from 2005 to 2020, and their Settings are shown in Table S2. It should be noted that in order to match the development path of PEP to protect ecological land, additional restrictions and corrections were made to the LUCC transfer matrix, as shown in Table S3.

Table S2 LUCC Transfer Matrix and Neighborhood Weight

| Land use | Cultivated land | Forest | Meadow | Water | Construction land | Unused land |
| --- | --- | --- | --- | --- | --- | --- |
| Cultivated land | 1 | 1 | 1 | 1 | 1 | 1 |
| Forest | 1 | 1 | 1 | 1 | 1 | 0 |
| Meadow | 1 | 1 | 1 | 0 | 1 | 1 |
| Water | 1 | 1 | 0 | 1 | 1 | 1 |
| Construction land | 1 | 1 | 0 | 1 | 1 | 0 |
| Unused land | 1 | 0 | 1 | 0 | 1 | 1 |
| Neighbour Weight | 0.279310411 | 0.574336726 | 0.000108589 | 0.037656938 | 0.108569201 | 1.81344E-05 |

Table S3 LUCC Transfer Matrix and Neighborhood Weight under PEP Scenario

| Land use | Cultivated land | Forest | Meadow | Water | Construction land | Unused land |
| --- | --- | --- | --- | --- | --- | --- |
| Cultivated land | 1 | 1 | 1 | 0 | 1 | 1 |
| Forest | 0 | 1 | 0 | 1 | 0 | 0 |
| Meadow | 0 | 1 | 1 | 0 | 0 | 1 |
| Water | 0 | 0 | 0 | 1 | 0 | 0 |
| Construction land | 1 | 1 | 0 | 0 | 1 | 0 |
| Unused land | 0 | 1 | 1 | 0 | 0 | 1 |
| Neighbour Weight | 0.279310411 | 0.574336726 | 0.000108589 | 0.037656938 | 0.108569201 | 1.81344E-05 |

**S6 Landscape Pattern Indices**

The landscape pattern Indices selected in this paper is mainly divided into four aspects: fragmentation, shape, aggregation and diversity, with reference to the research of Bao and Jiao (Bao and Yang, 2022b; Jiao et al., 2019). The specific index formula and description are shown in Table S9.

Table S9 Description of Landscape Pattern Indices

| **Landscape pattern indices** | | **Formula** |
| --- | --- | --- |
| fragmentation | LPI | $LPI=\frac{a_{\max}}{A}\times100$ |
|  | DIVISION | $\text{p}_{\text{i}}\text{=}\frac{\text{D}_{\text{ij}}}{\text{M}_{\text{ij}}}$ |
| shape | LSI | $\text{LSI=}\frac{\text{0.25}\int_{\text{i}}^{\text{n}} \text{e}_{\text{ij}}^{\text{*}}}{\sqrt{\text{TA}}}$ |
|  | COHESION | $COHESION=\left[ 1-\frac{\sum_{j=1}^{m} p_{ij}}{\sum_{j=1}^{m} p_{ij}\sqrt{a_{ij}}} \right]\left[ 1-\frac{1}{\sqrt{A}} \right]^{-1}\times100$ |
| aggregation | LJI | $LJI=\frac{-\sum_{i=1}^{m} \sum_{k=i+1}^{m} \left[ \left( \frac{e_{ik}}{E} \right)\cdot\ln\left( \frac{e_{ik}}{E} \right) \right]}{\ln\left( 0.5\left[ m\left( m-1 \right) \right] \right)}\times100$ |
|  | CONTAG | $\text{CONTAG=}\left[ \text{1+}\left( \int_{\text{i=}\text{1}}^{\text{n}} \int_{\text{j=}\text{1}}^{\text{n}} \left[ \left( \text{p}_{\text{i}} \right)\left( \frac{\text{f}_{\text{ij}}}{\int_{\text{i=1}}^{\text{n}} \text{f}_{\text{ij}}} \right) \right]\left[ \ln\left( \text{p}_{\text{i}} \right)\frac{\text{f}_{\text{ij}}}{\int_{\text{i=1}}^{\text{n}} \text{f}_{\text{ij}}} \right] \right)\text{÷}\text{2}\ln\left( \text{n} \right) \right]\text{×}\text{100}$ |
| diversity | SHDI | $\text{SHDI=-}\int_{\text{i=}\text{1}}^{\text{n}} \left( \text{p}_{\text{i}}\ln\text{p}_{\text{i}} \right)$ |
|  | SHEI | $\text{SHEI=}\frac{\text{-}\int_{\text{i=1}}^{\text{n}} \left( \text{p}_{\text{i}}\ln\text{p}_{\text{i}} \right)}{\ln\left( \text{n} \right)}$ |

Reference：

Bao, S., Yang, F., 2022a. Influences of Climate Change and Land Use Change on the Habitat Suitability of Bharal in the Sanjiangyuan District, China. International Journal of Environmental Research and Public Health 19.

Bao, S., Yang, F., 2022b. Spatio-Temporal Dynamic of the Land Use/Cover Change and Scenario Simulation in the Southeast Coastal Shelterbelt System Construction Project Region of China. Sustainability 14.

Cai, C., Ding, S., Shi, Z., Huang, L., Zhang, G., 2000. Study of Applying USLE and Geographical Information System IDRISI to Predict Soil Erosion in Small Watershed. Journal of Soil and Water Conservation 14, 19-24.

China, M.o.E.a.E.o.t.P.s.R.o., 2021. Technical specification for investigation and assessment of national ecological status——Ecosystem services assessment, <https://www.mee.gov.cn/>.

Ding, Y., Wang, L., Gui, F., Zhao, S., Zhu, W., 2022. Ecosystem Carbon Storage in Hangzhou Bay Area Based on InVEST and PLUS Models. Environmental Science 44, 3343-3352.

Jiao, M., Hu, M., Xia, B., 2019. Spatiotemporal dynamic simulation of land-use and landscape-pattern in the Pearl River Delta, China. Sustainable Cities and Society 49.

Liu, A., Wang, J., Liu, Z., 2009. Remote Sensing Quantitative Monitoring of Soil Erosion in Three Gorges Reservoir Area: a GIS/RUSLE - based Research. Journal of Natural Disasters 18, 25-30.

Nie, W., Xu, B., Yang, F., Shi, Y., Liu, B., Wu, R., Lin, W., Pei, H., Bao, Z., 2023. Simulating future land use by coupling ecological security patterns and multiple scenarios. Sci Total Environ 859, 160262.

Peng, S., Ding, Y., Wen, Z., Chen, Y., Cao, Y., Ren, J., 2017. Spatiotemporal change and trend analysis of potential evapotranspiration over the Loess Plateau of China during 2011–2100. Agricultural and Forest Meteorology 233, 183-194.

Sharp, R., Tallis, H.T., Ricketts, T., Guerry, A.D., Wood, S.A., ChaplinKramer, R., Nelson, E., Ennaanay, E., Wolny, S., Olwero, N., 2016. InVEST +VERSION+ User’s Guide. The natural capital project, Stanford University.

Yue, T., Yin, S., Xie, Y., Yu, B., Liu, B., 2022. Rainfall erosivity mapping over mainland China based on high-density hourly rainfall records. Earth System Science Data 14, 665-682.

Zerrouqi, Z., Sbaa, M., Oujidi, M., Elkharmouz, M., Bengamra, S., Zerrouqi, A., 2008. Assessment of cement’s dust impact on the soil using principal component analysis and GIS. International Journal of Environmental Science & Technology 5, 125-134.

Zhang, L., Dawes, W.R., Walker, G.R., 2001. Response of mean annual evapotranspiration to vegetation changes at catchment scale. Water Resources Research 37, 701-708.

Zhu, C., Zhang, X., Zhou, M., He, S., Gan, M., Yang, L., Wang, K., 2020. Impacts of urbanization and landscape pattern on habitat quality using OLS and GWR models in Hangzhou, China. Ecological Indicators 117.

Zou, T., Yoshino, K., 2017. Environmental vulnerability evaluation using a spatial principal components approach in the Daxing’anling region, China. Ecological Indicators 78, 405-415.
